# Sciatic nerve stimulation alleviates neuropathic pain and associated neuroinflammation in the dorsal root ganglia in a rodent model

**DOI:** 10.1186/s12967-024-05573-1

**Published:** 2024-08-14

**Authors:** Chia-En Wong, Wentai Liu, Chi-Chen Huang, Po-Hsuan Lee, Han-Wei Huang, Yu Chang, Hsin-Tien Lo, Hui-Fang Chen, Li-Chieh Kuo, Jung-Shun Lee

**Affiliations:** 1 Division of Neurosurgery, Department of Surgery, College of Medicine, National Cheng Kung University Hospital, National Cheng Kung University, No. 138, Sheng-Li Road, Tainan, 70428 Taiwan; 2 Department of Bioengineering, University of California, Los Angeles, CA USA; 3 California NanoSystems Institute, University of California, Los Angeles, CA USA; 4 Department of Electrical and Computer Engineering, Los Angeles, CA USA; 5 Brain Research Institute, University of California, Los Angeles, CA USA; 6 Department of Neurology, College of Medicine, National Cheng Kung University Hospital, National Cheng Kung University, Tainan, Taiwan; 7 Department of Cell Biology and Anatomy, College of Medicine, National Cheng Kung University, Tainan, Taiwan; 8 Department of Occupational Therapy, College of Medicine, National Cheng Kung University, Tainan, Taiwan; 9 Institute of Basic Medical Sciences, College of Medicine, National Cheng Kung University, Tainan, Taiwan

**Keywords:** Macrophage, Neuropathic pain, Satellite glial cell, Sciatic nerve stimulation

## Abstract

**Background:**

Satellite glial cells (SGCs) in the dorsal root ganglia (DRG) play a pivotal role in the formation of neuropathic pain (NP). Sciatic nerve stimulation (SNS) neuromodulation was reported to alleviate NP and reduce neuroinflammation. However, the mechanisms underlying SNS in the DRG remain unclear. This study aimed to elucidate the mechanism of electric stimulation in reducing NP, focusing on the DRG.

**Methods:**

L5 nerve root ligation (NRL) NP rat model was studied. Ipsilateral SNS performed 1 day after NRL. Behavioral tests were performed to assess pain phenotypes. NanoString Ncounter technology was used to explore the differentially expressed genes and cellular pathways. Activated SGCs were characterized in vivo and in vitro. The histochemical alterations of SGCs, macrophages, and neurons in DRG were examined in vivo on post-injury day 8.

**Results:**

NRL induced NP behaviors including decreased pain threshold and latency on von Frey and Hargreaves tests. We found that following nerve injury, SGCs were hyperactivated, neurotoxic and had increased expression of NP-related ion channels including TRPA1, Cx43, and SGC-neuron gap junctions. Mechanistically, nerve injury induced reciprocal activation of SGCs and M1 macrophages via cytokines including IL-6, CCL3, and TNF-α mediated by the HIF-1α-NF-κB pathways. SNS suppressed SGC hyperactivation, reduced the expression of NP-related ion channels, and induced M2 macrophage polarization, thereby alleviating NP and associated neuroinflammation in the DRG.

**Conclusions:**

NRL induced hyperactivation of SGCs, which had increased expression of NP-related ion channels. Reciprocal activation of SGCs and M1 macrophages surrounding the primary sensory neurons was mediated by the HIF-1α and NF-κB pathways. SNS suppressed SGC hyperactivation and skewed M1 macrophage towards M2. Our findings establish SGC activation as a crucial pathomechanism in the gliopathic alterations in NP, which can be modulated by SNS neuromodulation.

**Supplementary Information:**

The online version contains supplementary material available at 10.1186/s12967-024-05573-1.

## Background

Neuropathic pain (NP), characterized by mechanical allodynia and thermal hypersensitivity, results from a primary lesion of the nervous system. In NP animal models including rats with L5 nerve root ligation (NRL), pain behaviors are accompanied by abnormal activation of pain-conducting pathways [1].

The dorsal root ganglia (DRG), which contain the primary sensory neurons and supporting satellite glial cells (SGCs), are responsible for generating and transmitting sensory information from the periphery to the central nervous system (CNS). SGCs are abundant in DRG, where they encircle the cell bodies of primary sensory neurons [2]. The unique organization of SGCs and neurons allows for close interactions of these cells as neuron–glial units, which enable SGCs to regulate neuronal homeostasis in the DRG [3]. Nonetheless, the SGC–neuron homeostasis can be dysregulated upon nerve injury and NP [4]. Following peripheral nerve injury, NP behaviors including mechanical and thermal hypersensitivities are accompanied by activation of SGCs and release of inflammatory mediators by SGCs in the corresponding DRG [5, 6]. These changes in SGCs have been shown to contribute to the formation and maintenance of NP by augmenting neuronal activity and promoting neuroinflammation in various NP models as well as in humans [7–9].

In our previous work, we demonstrated the therapeutic potential of 20-Hz sciatic nerve stimulation (SNS) in alleviating mechanical allodynia and thermal hypersensitivity following L5 nerve root injury [10]. Furthermore, SNS was able to reduce the neuroinflammatory response in the spinal cord, manifested by the hyperactivation of superficial spinal cord dorsal horn neurons, elevated expression of inflammatory mediators such as nuclear factor kappa-light-chain-enhancer of activated B cells (NF-κB), tumor necrosis factor (TNF)-α, interleukin (IL)-1β, and IL-6, activation of spinal cord astrocytes and microglia [10–12]. However, the specific effect of SNS on the DRG remains unclear. Since the DRG serves as the gateway for peripheral-to-central pain transmission and inflammatory mediators, e.g., colony-stimulating factor 1 (CSF1) transported from the injured primary sensory neurons to the spinal cord, play a critical role in the central sensitization of NP, understanding the mechanisms of SGC hyperactivation and associated neuroinflammation in the DRG potentially lead to innovative treatment strategies for alleviating NP [13–15]. Moreover, a comprehensive investigation into the mechanisms of SNS in the DRG can improve our understanding and the application of SNS and benefit patients suffering from NP.

To address these knowledge gaps, the present study aimed to elucidate the mechanism of the SNS, focusing on the DRG in an NP model of L5 NRL rats. We characterized the histomorphology and molecular alterations of DRG SGCs following NRL and evaluated how these changes in SGCs contribute to NP. Moreover, we investigated the effect of SNS on the neuroinflammation in SGCs and primary sensory neurons following NRL.

## Methods

### Ethics statements and animals

All surgical interventions and perioperative care were performed according to the guidelines of the Institute of Animal Use and Care Committee. Adult Sprague–Dawley male rats, aged 8 weeks and weighing 250–300 g, were obtained from BioLASCO (Nangang, Taipei, Taiwan) and housed at 25 ± 2 °C under a 12-h light–dark cycle. Efforts were made to reduce the number of animals used and to minimize animal suffering.

### L5 NRL model

Surgical procedures for L5 NRL were performed as previously described [10, 16]. The rats were anesthetized using Zoletil^®^ 50 (40 mg/kg; Virbac, Carros, France) via intraperitoneal injection and placed in the prone position. Using a paramedian incision, the paraspinal muscles were split and gently retracted to expose the transverse process, which was removed to expose the L5 nerve. The L5 nerve root was ligated using 4–0 non-absorbable sutures with three surgical ties. Wound closure was performed in a layer-by-layer manner. After NRL, a heating pad was used to maintain the rats’ temperature at 37 °C until recovery from anesthesia.

### Sciatic nerve stimulation

**Fig. 1:**
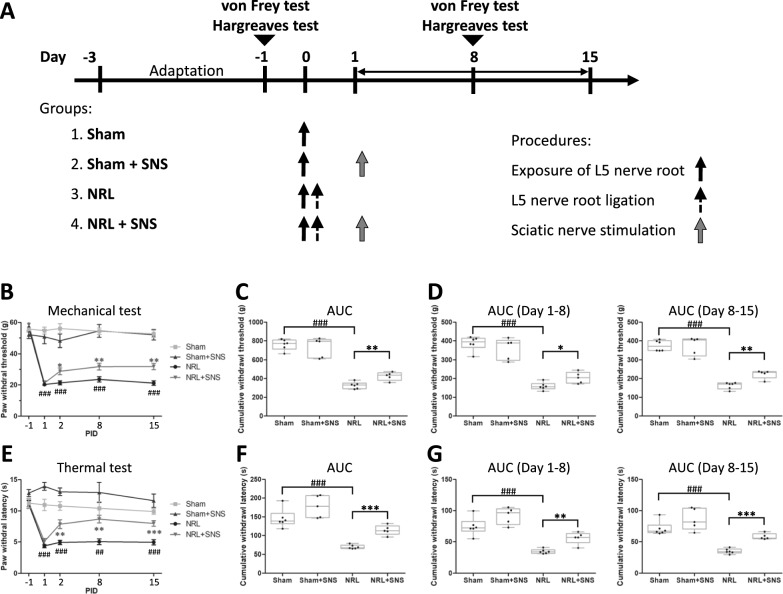
Study protocol and animal pain behavioral experiments. **A** The Sham group (group 1) with the L5 nerve root exposed but not ligated was used as the control. In the sciatic nerve stimulation (SNS) groups (groups 2 and 4), the SNS electrode was placed distal to the joint of the L4, L5, and L6 nerve roots at 1-day after nerve root ligation (NRL), and electrical stimulation was performed with 20-Hz for 1 h. In the NRL groups (groups 3 and 4), the L5 nerve root was exposed and ligated. Behavioral tests, including the von Frey and Hargreaves tests, performed 1 day before NRL and on post-injury days (PIDs) 1 (before electric stimulation), 2, 8, and 15. **B** The paw withdrawal thresholds on the ipsilateral side to NRL and SNS (N = 5–6). **C** The area under the curve (AUC) calculated as the cumulative withdrawal threshold. **D** The AUCs of the paw withdrawal thresholds on PIDs 1–8 and 8–15. **E** The paw withdrawal latencies on the ipsilateral side to NRL and SNS (N = 5–6). **F** The AUC calculated as the cumulative withdrawal latency. **G** The AUCs of the paw withdrawal latencies on PIDs 1–8 and 8–15. Data are expressed as mean ± standard deviation (SD). ^##^p < 0.01 compared with the Sham group, ^###^p < 0.001 compared with the Sham group, *p < 0.05 compared with the NRL group, **p < 0.01 compared with the NRL group, ***p < 0.001 compared with the NRL group

Four sets of animal experiments were performed (Fig. 1A): Sham (exposure of the L5 nerve root without ligation, *N* = 6), Sham + SNS (*N* = 5), NRL (L5 NRL, *N* = 6), and NRL + SNS (*N* = 5). SNS was performed on the day following NRL or Sham surgery (PID 1) in a 1-h single session.

The stimulated sciatic nerve segment was located ipsilateral to the ligated L5 nerve root and distal to the L4, L5, and L6 nerve roots. SNS was delivered via custom-made bipolar electrodes connected to a nerve conduction stimulator (ISIS Xpress; Medizintechnik GmbH, Tuttlingen, Germany). The delivered stimulus consisted of 2-s pulse trains with a waveform of 20 Hz, biphasic, 200-μs square wave pulses. The 2-s pulse trains were separated at 8-s intervals. The intensity ranged from 0.1 to 1 mA. Motor responses were used to confirm the delivery of electrical stimulation. Subsequently, the stimulation intensity was selected as the maximal intensity that did not trigger motor responses. These parameters were adapted from previous studies that demonstrated that such stimuli activated all nerve fiber types [17].

### Behavioral tests

Three days before NRL surgery, the rats were introduced to the behavioral testing environment daily for adaptation. Before behavioral tests, the rats were placed in a testing environment for 30 min. On the day before surgery and at 1, 2, 8, and 15 days after surgery, the plantar surfaces of both ipsilateral and contralateral hind paws were subjected to mechanical touch sensitivity tests (von Frey test) and thermal sensitivity tests (Hargreaves test). In the von Frey tests, electrical von Frey tips (BIO-EVF5; Bioseb, Vitrolles, France) were gently applied to the plantar surface of the rats, and force was slowly exerted until the rats withdrew, flicked, or licked their paws. The maximum force was recorded automatically. Each rat was tested five times at 15-s intervals. The Hargreaves test was performed using a Plantar Test Apparatus (Ugo Basile, Comerio, Italy). The intensity of the infrared heat source (50 W) was adjusted such that naïve rats had withdrawal latencies of 9–12 s. The heat source was focused on the plantar surface of the hind paw, and the time to paw withdrawal was recorded. Each hind paw was tested three times, separated by 2-min intervals. On PID1, pain tests were performed before SNS to evaluate changes following NRL alone.

### Western blot analysis

The L5 DRG was freshly collected and homogenized in T-PER reagent buffer with a protease inhibitor mixture (Thermo Fisher Scientific, Waltham, MA, USA). Tissue homogenates were centrifuged at 12,000 rpm for 15 min at 4 °C. The supernatant was collected. A protein assay kit (Micro BCA™; Thermo Fisher Scientific) quantified the protein concentrations.

Protein extracts (30 μg) were resuspended in a loading buffer, subjected to polyacrylamide gel electrophoresis and transferred onto nitrocellulose membranes. The membranes were subsequently blocked with 5% non-fat milk in Tris-buffered saline with Tween-20 (20-mM Tris base, 130-mM NaCl, 0.1% Tween-20), and incubated overnight at 4 °C with the following primary antibodies: anti-β-actin (A5441, 1:10000; Sigma-Aldrich, Kawasaki, Kanagawa, Japan), anti-NF-κB (#8242, 1:2000; Cell Signaling Technology, Danvers, MA, USA), anti-TRPA1 (ACC-037, 1:1000; Alomone Labs, Jerusalem, Israel), and anti-HIF-1α (sc-10790, 1:2000; Santa Cruz). Then, the membranes were washed and incubated with horseradish peroxidase-conjugated secondary antibodies (Jackson ImmunoResearch Inc., West Grove, PA, USA) at room temperature for 1 h. Western blot analysis was performed using an enhanced chemiluminescence detection kit (WBKLS000; Merck Millipore, Merck KGaA, Burlington, MA, USA) and visualized using a luminescence imaging system (Azure Biosystems Dublin, California, USA). Protein levels were normalized to β-Actin and analyzed using ImageJ software (National Institutes of Health, Bethesda, MD, USA). All experiments were performed in triplicate.

### ELISA analysis

The L5 DRG was freshly collected and homogenized in T-PER reagent buffer with a protease inhibitor mixture (Thermo Fisher Scientific, Waltham, MA, USA) according to the manufacturer’s instructions. Tissue lysates were collected as previously described. Protein extracts were analyzed using rat IL-6 (Ab234570; Abcam, Cambridge, UK), CCL3 (Ab242242; Abcam), and TNF-α ELISA kits (Ab236712; Abcam), according to the manufacturer’s instructions.

### Immunofluorescence staining

After anesthesia, transcardial perfusion using ice-cold saline followed by 4% paraformaldehyde in 0.1-M phosphate buffered saline (PBS) was performed. The L5 DRG tissues were harvested and post-fixed in 4% paraformaldehyde, immersed in PBS with 30% sucrose for cryoprotection at room temperature for 48 h, and embedded in optimal cutting temperature compound in liquid nitrogen. Twenty-μm cryosections of the DRG were cut using a cryostat (CM1950; LEICA, Wetzlar, Germany) at −20 °C for further immunofluorescence.

Sections were permeabilized and blocked with 10% normal goat serum (prepared in PBS supplemented with 0.1% bovine serum albumin and 0.1% Triton X-100) for 20 min. Next, the sections were incubated overnight at 4 °C with the following primary antibodies: anti-GFAP (G3893, 1:500; Sigma-Aldrich), anti-S100A10 (11250-1-AP, 1:200; Proteintech), anti-NeuN (MAB377, 1:200; Merck), anti-NeuN (MABN140, 1:200; Merck), anti-TRPA1 (ACC-037 1:200; Alomone Labs), anti-Cx43 (610062, 1:200; BD Biosciences, Franklin Lakes, NJ, USA), anti-Iba1 (GTX100042, 1:200; GexeTex, Zeeland, MI, USA), anti-iNOS (GTX636531, 1:500; GeneTex), anti-CD206 (A8301, 1:200; ABclonal, Woburn, MA, USA), and anti-ATF3 (sc-518032, 1:200; Santa Cruz). Then, the sections were incubated with fluorescent secondary antibodies (Thermo Fisher Scientific) for 1 h at room temperature and mounted using media with 4′,6-diamidino-2-phenylindole (Abcam). Fluorescent images were obtained using a fluorescence microscope (M568E; Nikon, Minato City, Tokyo, Japan). All experiments were performed in triplicate.

### NanoString nCounter analysis for gene profiling

For the NanoString nCounter analysis, total RNA was extracted and quantified following the manufacturer’s protocol (TRIzol; Thermo Fisher Scientific). mRNAs were hybridized using a NanoString nCounter neuroinflammation panel of 770 barcoded probes (NanoString Technologies, Seattle, WA, USA). Probe-tagged fluorescent barcodes were counted using a digital analyzer. Three biological replicates were performed for each condition. Standard NanoString protocols were used according to the manufacturer’s instructions. The detected mRNA probe counts were analyzed using NanoString nSolver software to identify DEGs.

### Electron microscopy

TEM was used to examine gap junctions between SGCs and neurons. The excised DRGs were post-fixed for 45 min (15 min × 3) with 1% osmium tetroxide in PBS, dehydrated through a graded series of ethyl alcohol, cleared with QY-1, and embedded in an epoxy resin mixture. Ultrathin sections (100 nm) were obtained using a diamond knife. The stained samples were examined under a transmission electron microscope (JEM-1400; JOEL, Peabody, MA, USA).

### Cell cultures and conditioned media treatment

For SGC culture, DRGs were isolated from neonatal P0 rats, digested with 0.1% collagenase for 1 h, 0.25% trypsin for 30 min, and inactivated in serum containing medium. Next, the cells were pelleted by centrifugation at 500 × g for 2 min, the supernatant was aspirated, and the pellet was resuspended in the culture medium. Cultures were maintained in Dulbecco's modified eagle medium (DMEM) plus 10% of fetal bovine serum and penicillin (50 U/ml)/streptomycin (50 mg/ml) at 37 °C with 5% carbon dioxide (CO_2_). For LPS treatment and conditioned medium collection, primary SGC culture at 5–7 DIVs was treated with LPS (*Escherichia coli* 026:B6) at 0.5 and 2.0 µg/ml for 24 h. After LPS treatment, fresh culture medium was added, and the medium was collected 48 h later as the SGC-conditioned medium.

Before primary DRG neuron culture, glass coverslips were coated with 1-mg/ml poly-d-lysine at room temperature for 20 min. Neonatal P0 rats 0 were sacrificed and DRG tissues were triturated for disaggregation, filtered, and plated in neurobasal medium plus B27 serum-free supplement and antibiotics (50-U/ml penicillin and 50-μg/ml streptomycin) at 37 °C with 5% CO_2_. For conditioned medium treatment, primary DRG neurons at 12–14 DIVs were used, and the medium was replaced by DMEM plus 10% of fetal bovine serum (control medium), SGC-conditioned medium (0.5 µg/ml LPS treated), SGC-conditioned medium (2.0 µg/ml LPS treated), and DMEM plus 10% of fetal bovine serum and 2.0-µg/ml LPS for 24 h. Cell viability assays and immunofluorescence staining were then performed. For immunofluorescence, cells were washed with PBS and fixed with 4% PFA for 20 min. All experiments were performed in triplicate.

### Cell viability assay

Primary neurons were seeded at 3.0 × 10^3^ cells per well in a 24-well plate and maintained for 12–14 DIVs. Following treatment with the control medium, SGC-conditioned medium (0.5 µg/ml LPS treated), SGC-conditioned medium (2.0 µg/ml LPS treated), and DMEM plus 10% of fetal bovine serum and 2.0-µg/ml LPS for 24 h, the media were removed and replaced by a 50 mg/mL 3-(4, 5-dimethylthiazol-2-yl) − 2, 5-diphenyltetrazolium bromide (MTT) at tenfold dilution in fresh media for 3 h at 37 °C. The MTT was aspirated, followed by the addition of a dissolving agent, dimethyl sulfoxide, and incubated for 15 min. Optical density values were measured at 570 nm using a spectrophotometer to calculate cell viability. All experiments were performed in triplicates.

### Statistical analysis

Continuous variables are expressed as means and standard deviations. Comparisons between two groups were performed using Student’s *t*-test. Data from three or more groups were compared using one-way analysis of variance with Bonferroni’s post-hoc test. The regression analyses were performed using a linear regression model. Differences were considered statistically significant at p < 0.05.

## Results

### SNS alleviates mechanical allodynia and thermal hypersensitivity in L5 NRL rats.

To evaluate the effect of SNS, we first investigated the mechanical (von Frey) and thermal (Hargreaves) pain behaviors in L5 NRL rats (Fig. 1A). Compared with the Sham group, the NRL group exhibited significantly lower paw withdrawal thresholds from post-injury days (PIDs) 1 (54.8 ± 5.4 g vs 20.3 ± 1.9 g, p < 0.001), 2 (56.1 ± 7.6 g vs 21.4 ± 2.9 g, p < 0.001), 8 (54.3 ± 5.0 g vs 23.6 ± 3.8 g, p < 0.001), and 15 (52.7 ± 5.6 g vs 21.2 ± 3.7 g, p < 0.001) (Fig. 1B). Similarly, paw withdrawal latencies were also lower in NRL group compared to Sham in PID 1 (11.0 ± 2.2 s vs 4.4 ± 0.7 s, p < 0.001), 2 (10.8 ± 1.7 s vs 4.9 ± 0.8 s, p < 0.001), 8 (10.4 ± 2.6 s vs 5.1 ± 1.2 s, p = 0.008), and 15 (9.9 ± 1.1 vs 5.0 ± 1.0 s, p < 0.001) (Fig. 1E). After SNS, rats in the NRL + SNS group had increased paw withdrawal thresholds on PIDs 2 (21.4 ± 2.9 vs 28.8 ± 4.4, p = 0.046), 8 (23.6 ± 3.8 vs 31.8 ± 4.8, p = 0.009), and 15 (21.2 ± 3.7 vs 3.18 ± 4.5, p = 0.003) compared with rats in the NRL group (Fig. 1B, E). Similarly, the latencies of NRL + SNS rats in the thermal tests were also increased on PIDs 2 (4.9 ± 0.8 s vs 7.9 ± 1.6 s, p = 0.009), 8 (5.1 ± 1.2 s vs 8.7 ± 1.4 s, p = 0.002), and 15 (5.0 ± 1.0 s vs 8.0 ± 1.0 s, p < 0.001) compared with NRL rats. The differences in paw withdrawal thresholds and latencies between the Sham and Sham + SNS groups were not significant.

We calculated cumulative paw withdrawal thresholds (Fig. 1C, D). L5 NRL resulted in significantly decreased ipsilateral cumulative withdrawal thresholds overall (761.1 ± 55.7 g vs 327.2 ± 35.0 g, p < 0.001), and in both the first (386.6 ± 37.4 g vs 163.1 ± 19.9 g, p < 0.001) and second weeks (374.5 ± 27.9 g vs 162.3 ± 17.8 g, p < 0.001) after injury and these decreases wase ameliorated by SNS (overall: 327.2 ± 35.0 g vs 423.6 ± 47.8 g, p = 0.009; first week: 163.1 ± 19.9 g vs 200.9 ± 30.2 g, p = 0.047; second week: 162.3 ± 17.8 g vs 222.7 ± 24.4 g, p = 0.002). Similarly, L5 NRL resulted in significantly lower ipsilateral cumulative withdrawal latencies in the first 2 weeks after injury (Fig. 1D–F) (overall: 145.5 ± 25.2 s vs 69.6 ± 5.2, p < 0.001; first week: 74.4 ± 14.4 s vs 34.6 ± 3.6 s, p < 0.001; second week: 71.1 ± 11.2 s vs 35.0 ± 4.0 s, p < 0.001), and the decreases were ameliorated by SNS (overall: 69.6 ± 5.2 s vs 114.4 ± 13.2 s, p < 0.001; first week: 34.6 ± 3.6 s vs 56.1 ± 9.6, p = 0.005; second week: 35.0 ± 4.0 s vs 58.3 ± 4.8 s, p < 0.001). The mechanical and thermal behavior of the contralateral paw remained unchanged (Supplementary Figure S1).

### SNS alleviated L5 NRL-induced primary sensory neuron injury in DRG

**Fig. 2:**
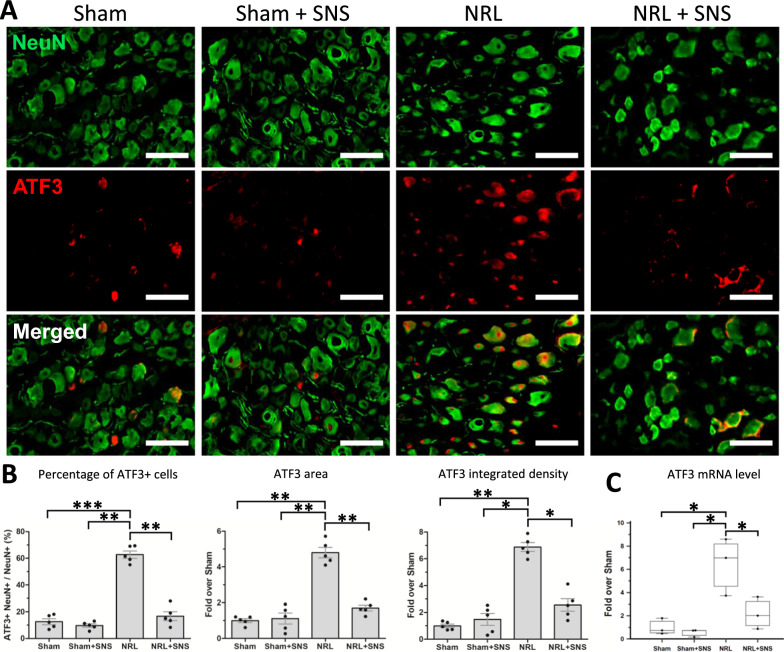
SNS alleviated L5 NRL-induced injury of the primary sensory neurons. **A** Immunofluorescence staining of NeuN (green) and activating transcription factor 3 (ATF3) (red) on post-injury day (PID 8) (N = 5). **B** The percentages of ATF3 neurons, ATF3 area, and integrated density of M2 quantified using ImageJ software. **C** The messenger RNA levels of ATF3C on PID 8 quantified by NanoString nCounter. Data are expressed as mean ± standard deviation. Scale bars = 200 μm. *p < 0.05, **p < 0.01, ***p < 0.001

Next, we investigated whether the alterations in pain behavior were correlated with changes in the histomorphology of DRG primary sensory neurons. Immunofluorescence co-staining of NeuN and ATF3 was performed on PID 8 (Fig. 2A). The percentage of ATF3 + /NeuN + cells (12.6 ± 4.9% vs 62.7 ± 6.2%, p < 0.001), area (1.0 ± 0.2 fold vs 4.8 ± 0.7 fold, p = 0.005), and integrated density of ATF3 (1.0 ± 0.3 fold vs 6.9 ± 0.7 fold, p = 0.006) were significantly elevated in NRL rats compared with Sham rats. Moreover, these changes in ATF3 signaling were alleviated by SNS (percentage: 62.7 ± 6.2% vs 16.7 ± 7.3%, p = 0.005; area: 4.8 ± 0.7 fold vs 1.7 ± 0.4 fold, p = 0.008; density: 6.9 ± 0.7 fold vs 2.6 ± 1.0 fold, p = 0.032) (Fig. 2B). Quantification of mRNA expression of ATF3 in the DRG also showed an increase after NRL (1.0 ± 0.6 fold vs 6.4 ± 2.5 fold, p = 0.022), which was alleviated by SNS (6.4 ± 2.5 fold vs 2.0 ± 1.2 fold, p = 0.049) (Fig. 2C). Additionally, the mRNA expression levels of c-fos, CSF1, glutamate ionotropic receptor AMPA type subunit 1 (GRIA1), and GRIA4 in the DRG on PID 8 were all elevated in the NRL group compared with the Sham group, and these changes were alleviated by SNS (Supplementary Figure S2). Regression analyses showed that the levels of these injury markers, including ATF3, c-fos, GRIA1, and GRIA4, were significantly correlated with pain thresholds in the behavioral experiments (Supplementary Figure S3).

### Alterations in DRG gene expression profiles following NRL and SNS

Based on the pain behavior results and the associated injury of primary sensory neurons, we further explored the alterations in the DRG after NRL and SNS. Therefore, we used NanoString nCounter technology to perform a comprehensive analysis of changes in gene expression in the DRG following L5 NRL and SNS.

**Fig. 3:**
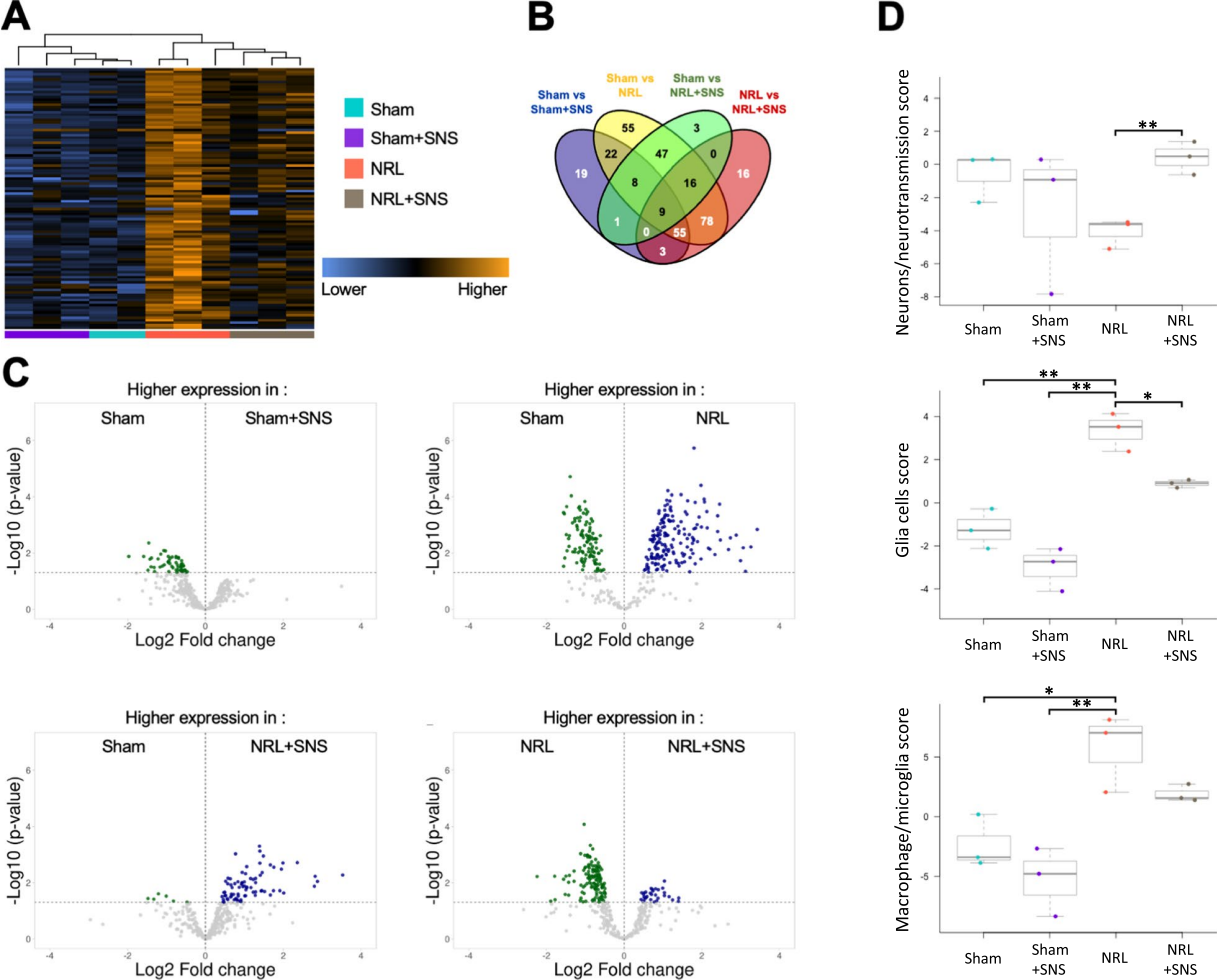
Differentially expressed genes (DEGs) and cellular pathways after NRL and SNS in the L5 DRG. The DEGs of the L5 DRG on post-injury day 8 were evaluated using a NanoString nCounter (N = 3). **A** Heatmap of DEGs in the L5 DRG between the four experimental groups. **B** Venn diagram showing the number of DEGs in the L5 DRG. **C** Volcano plots for differential gene expression. Scattered points represent genes; the x-axis represents the log2 fold change, and the y-axis represents the log(p-value). DEGs were discriminated based on a p-value at an α level of 0.05. Blue dots indicate significantly upregulated genes, whereas green dots indicate significantly downregulated genes. **D** Cellular pathway scores calculated based on nCounter analyses of the DEGs. *p < 0.05, **p < 0.01

Cluster analysis of gene expression from the nCounter panels separated the NRL and NRL + SNS groups from the Sham and Sham + SNS groups, indicating that nerve injury significantly changed the gene expression profile in the DRG (Fig. 3A). Specifically, we identified 332 differentially expressed genes (DEGs) (Fig. 3B), including 117, 290, 84, and 177 genes that were differentially expressed between Sham versus (vs.) Sham + SNS, Sham vs. NRL, Sham vs. NRL + SNS, and NRL vs. NRL + SNS groups, respectively (Fig. 3C–F). The DEGs are presented in Supplementary Table 1.

Further, pathway score analyses demonstrated that following NRL, the glial cell score (−1.2 ± 0.9 vs 3.4 ± 0.9, p = 0.004) and macrophage/microglia score (−2.0 ± 2.2 vs 5.8 ± 2.9, p = 0.034) were significantly elevated. The difference in neuron/neurotransmission score between the Sham and NRL groups was borderline significant (−0.4 ± 1.5 vs −4.0 ± 0.9, p = 0.050). Compared to the NRL group, NRL + SNS rats had decreased glial cell score (3.4 ± 0.9 vs 1.0 ± 0.2 p = 0.046) and increased neuron/neurotransmission score (−4.0 ± 0.9 vs 0.3 ± 1.0p = 0.007). Although there was a mild trend of decreased macrophage/microglia score in the NRL + SNS group compared to NRL, the difference was not significant (5.8 ± 2.9 vs 1.8 ± 0.6, p = 0.092).

### Modulatory effect of SNS on SGC activation and expression of ion channels and gap junctions in the SGCs in L5 NRL rats

**Fig. 4:**
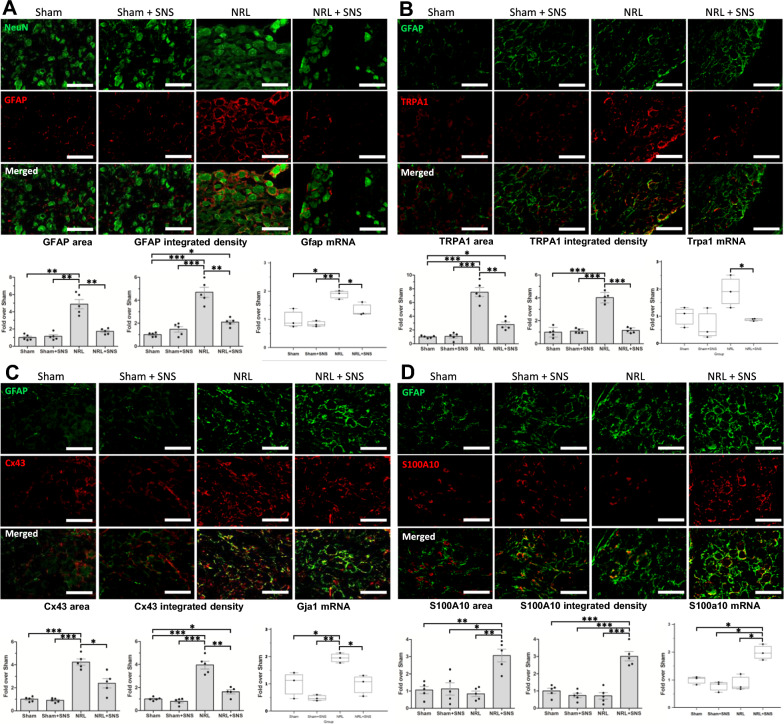
Activation SGCs and expression of ion channels and gap junctions in L5 DRG following NRL and SNS. Cryosections of the ipsilateral L5 DRG were obtained from the nerve root ligation rats on post-injury day (PID) 8 (N = 5). **A** Representative immunofluorescence staining of the SGC marker glial fibrillary acidic protein (GFAP) (red) and neuron marker NeuN (green) with quantification of relative area and integrated density of the GFAP signal. Gfap mRNA levels were quantified by nCounter analysis. **B** Immunofluorescence staining of GFAP (green) and transient receptor potential cation channel subfamily A member 1 (TRPA1) (red) with quantification of the TRPA1 area and integrated density on post-injury day 8. Trpa1 mRNA levels were quantified by nCounter analysis. **C** Immunofluorescence staining of NeuN (green) and connexin 43 (Cx43) (red) with quantification of the Cx43 area and integrated density. Gja1mRNA levels were quantified by nCounter analysis. **D** Immunofluorescence staining of NeuN (green) and S100A10 (red) with quantification of the S100A10 area and integrated density. S100a10 levels were quantified by nCounter analysis. Scale bars = 200 μm. *p < 0.05, **p < 0.01, ***p < 0.001

Given that the glial cell pathway score had the most significant alterations among all cell types following NRL and SNS, we hypothesized that SGCs in the DRG contributed to the pathogenesis of NP following NRL and might be modulated by SNS. Double immunofluorescence staining showed the signal of GFAP surrounded that of NeuN (Fig. 4A). This configuration indicates the glia-neuron units consisting of SGCs surrounding the primary sensory neurons in the DRG. Moreover, quantification of the GFAP signal revealed an increased area (1.0 ± 0.3 fold vs 4.9 ± 1.2 fold, p = 0.001) and integrated density (1.0 ± 0.2 fold vs 4.7 ± 1.0 fold, p < 0.001) following L5 NRL, which could be alleviated by SNS on PID 1 (area: 4.9 ± 1.2 fold vs 1.7 ± 0.3 fold, p = 0.005; integrated density: 4.7 ± 1.0 fold vs 2.1 ± 0.3 fold, p = 0.003). Additionally, quantification of gfap mRNA level showed an elevated gfap mRNA level following NRL (1.0 ± 0.4 fold vs 1.9 ± 0.2 fold, p = 0.041), and the change was alleviated by SNS (1.9 ± 0.2 fold vs 0.7 ± 0.7 fold, p = 0.048).

Previous studies have shown that the abnormal expression of ion channels in SGCs plays an essential role in NP formation [18–20]. Therefore, we investigated whether SNS affects the expression of NP-related channels in SGCs, including transient receptor potential cation channel subfamily A member 1 (TRPA1) and Cx43. Immunofluorescence co-staining of the SGC markers GFAP and TRPA1 on PID 8 in the DRG showed that the signals of TRPA formed ringed structures and overlapped with the GFAP signals (Fig. 4B), indicating that following L5 NRL, the expression of DRG TRPA1 was primarily in the SGCs. Furthermore, quantification of TRPA1 showed an increased area (1.0 ± 0.2 fold vs 7.5 ± 1.5 fold, p < 0.001) and integrated density (1.0 ± 0.4 fold vs 4.0 ± 0.5 fold, p < 0.001) following L5 NRL, which could be alleviated by SNS on PID 1 (area: 7.5 ± 1.5 fold vs 2.8 ± 9,eightfold, p = 0.007; integrated density: 4.0 ± 0.5 fold vs 1.2 ± 0.2 fold, p < 0.001). Likewise, the trpa1 mRNA level was significantly decreased in the NRL + SNS group compared to NRL (1.9 ± 0.6 fold vs 0.9 ± 0.1 fold, p = 0.044). Immunofluorescence staining of Cx43 revealed that following L5 NRL, the area (1.0 ± 0.2 fold vs 4.2 ± 0.6 fold, p < 0.001) and integrated density (1.0 ± 0.1 fold vs 3.9 ± 0.7 fold, p < 0.001) of Cx43 were increased. Application of SNS showed that the area (4.2 ± 0.6 fold vs 2.4 ± 0.9 fold, p = 0.025) and integrated density (3.9 ± 0.7 fold vs 1.6 ± 0.4 fold, p = 0.007) of Cx43 were decreased in the NRL + SNS group compared to NRL. Similarly, NRL increased Gja1 mRNA expression (1.0 ± 0.5 fold vs 1.9 ± 0.2 fold, p = 0.042), which was alleviated in the NRL + SNS group (1.9 ± 0.2 vs 1.0 ± 0.4 fold, p = 0.037) (Fig. 4C). Additionally, we investigated the expression of TRPV1 in the DRG. Immunofluorescence co-staining of the TRPV1 and NeuN showed that TRPV1 was primarily expressed in neurons and the difference of TRPV1 signal was not significant between the groups (Supplementary Figure S4).

Next, we investigated the alterations of S100A10, a marker of neuroprotective glia, in the DRG SGCs following NRL and SNS [21, 22]. Quantification of S100A10 immunofluorescence showed that the area of S100A10 in NRL + SNS group was significantly elevated compared to Sham (3.1 ± 0.8 vs 1.0 ± 0.5 fold, p = 0.003), Sham + SNS (3.1 ± 0.8 vs 1.1 ± 0.8 fold, p = 0.006), and NRL (3.1 ± 0.8 vs 0.8 ± 0.3 fold, p = 0.002). The integrated density of S100A10 was also significantly elevated compared to Sham (3.0 ± 0.6 vs 1.0 ± 0.3 fold, p < 0.001), Sham + SNS (3.0 ± 0.6 vs 0.7 ± 0.3 fold, p < 0.001), and NRL (3.0 ± 0.6 vs 0.7 ± 0.4 fold, p < 0.001). Likewise, S100A10 mRNA in NRL + SNS group was significantly increased compared to Sham (2.0 ± 0.3 vs 1.0 ± 0.2 fold, p = 0.014), Sham + SNS (2.0 ± 0.3 vs 0.8 ± 0.2 fold, p = 0.016), and NRL (2.0 ± 0.3 vs 1.3 ± 0.3 fold, p = 0.038) (Fig. 4D).

### The effect of SNS on SGC-neuron gap junctions in L5 NRL rats

**Fig. 5:**
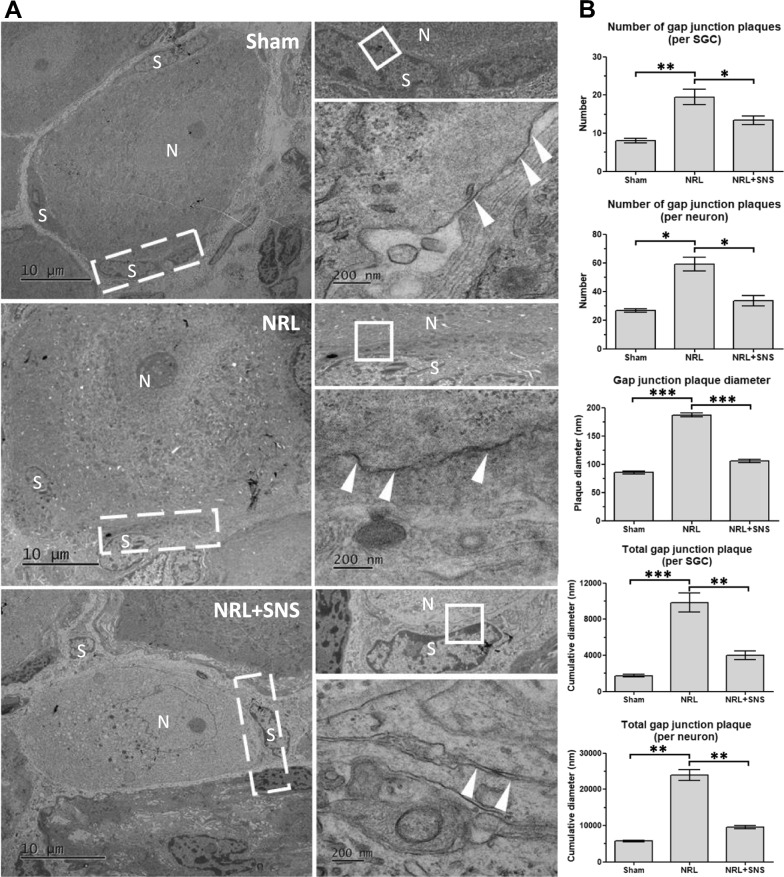
Modulatory effect of SNS on SGC-primary sensory neuron gap junctions in the DRGs in L5 NRL rats. **A** Transmission electron microscopy images of the L5 DRG showing SGC surrounding the primary sensory neurons. Magnified regions are indicated in white rectangles. Arrowheads indicate gap junctions between SGCs and sensory neurons. N: primary sensory neuron; S: SGCs. Scale bars = 10 μm and 200 nm. **B** Quantifications of glia-neuron gap junction plaques (N = 3). Data are expressed as mean ± standard deviation. *p < 0.05, **p < 0.01, ***p < 0.01

Next, to investigate whether SGC–neuron communication was altered in NP and SNS, Transmission electron microscopy (TEM) was used to examine the gap junctions between SGCs and neurons (Fig. 5A). The number, diameter, and cumulative diameter of the gap junction plaques between primary sensory neurons and surrounding SGCs were quantified (Fig. 5B). NRL increased gap junction plaques per SGC (8.0 ± 1.6 vs 19.5 ± 4.7, p = 0.002), per neuron (26.7 ± 0.7 vs 59.0 ± 4.9, p = 0.011), plaque diameter (85.5 ± 5.4 nm vs 186.9 ± 9.7 nm, p < 0.001), cumulative diameter per SGC (1731.3 ± 242.1 nm vs 9844.5 ± 1331.3 nm, p < 0.001), and cumulative diameter per neuron (5771.0 ± 279.3 nm vs 23,936.7 ± 1748.0 nm, p = 0.001). These alterations in the SGC–neuron gap junctions were alleviated by SNS (plaques per SGC: 19.5 ± 4.7 vs 13.4 ± 2.8, p = 0.032; per neuron: 59.0 ± 4.9 vs 33.5 ± 2.5, p = 0.025; plaque diameter: 186.9 ± 9.7 nm vs 105.6 ± 6.9 nm, p < 0.001; cumulative diameter per SGC: 9844.5 ± 1331.3 nm vs 3990.6 ± 577.4 nm, p = 0.006; cumulative diameter per neuron: 23,936.7 ± 1748.0 nm vs 9572.0 ± 480.6 nm, p = 0,002). These results demonstrate that the ion channels of SGCs are involved in the formation of NP and that SNS indeed modulates these ion channels and gap junctions.

### Activated SGCs were neurotoxic and induced loss of synapses and neuronal deaths in vitro.

Normally, the SGCs maintain homeostasis and promote neuronal survival [3]. To determine the effect of activated SGCs on neuron survival, primary rat DRG neurons were cultured for 12–14 days in vitro (DIVs) and exposed to SGC-conditioned medium for 24 h, and their viability was measured. The SGC-conditioned medium was obtained from cultured SGCs treated with LPS, which showed elevated TRPA1 and TNF immunofluorescence intensities (Supplementary Figure S5).

**Fig. 6:**
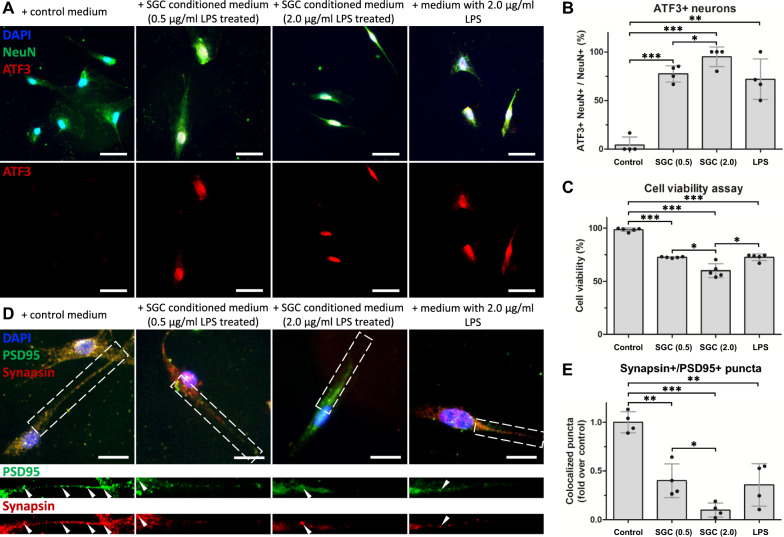
In vitro characterization of activated SGCs. **A** Immunofluorescence staining of NeuN (green) and activating transcription factor 3 (ATF3) (red) in primary rat DRG neurons treated with the control medium, SGC-conditioned medium, and lipopolysaccharide (LPS). Scale bars = 20 μm. **B** Quantification of ATF3-positive neurons (N = 4). **C** Cell viability assay results of primary neurons treated with the control medium, SGC-conditioned medium, and LPS (N = 5). **D** Immunofluorescence staining of PSD95 (green) and synapsin (red) in primary neurons treated with the control medium, SGC-conditioned medium, and LPS. Magnified regions are indicated in dashed rectangles. Scale bars = 5 μm. **E** Quantification of synapsin/PSD95 co-localized puncta (N = 4). Data are expressed as mean ± standard deviation. *p < 0.05, **p < 0.01, ***p < 0.01

Cultured neurons showed markedly increased activating transcription factor 3 (ATF3) immunofluorescence percentage after 24 h in the SGC-conditioned medium previously exposed with increasing concentrations of LPS (Control vs 0.5 μg/ml: 4.2 ± 8.3% vs 77.5 ± 8.4%, p < 0.001; Control vs 2.0 μg/ml: 4.2 ± 8.3% vs 95.0 ± 10.0%, p < 0.001; Control vs LPS: 4.2 ± 8.3% vs 72.1 ± 20.8%, p = 0.004) (Fig. 6A, B). The cell viability assay revealed that activated SGCs were toxic to neurons (Control vs 0.5 μg/ml: 98.4 ± 1.6% vs 72.4 ± 0.6%, p < 0.001; Control vs 2.0 μg/ml: 98.4 ± 1.6% vs 59.9 ± 6.4%, p < 0.001; Control vs LPS: 98.4 ± 1.6% vs 72.4 ± 3.2%, p < 0.001) (Fig. 6C). SGC-conditioned medium obtained from SGCs previously treated with 2.0-μg/ml LPS was more toxic than that obtained from SGCs treated with 0.5-μg/ml LPS (ATF3: 77.5 ± 8.4% vs 95.0 ± 10.0%, p = 0.038; Cell viability: 72.4 ± 0.6% vs 59.9 ± 6.4%, p = 0.012). Moreover, SGC-conditioned medium obtained from SGCs previously treated with 2.0-μg/ml LPS was also more toxic than the control medium supplemented with 2.0-μg/ml LPS (Cell viability: 59.9 ± 6.4% vs 72.4 ± 3.2% p = 0.017). Next, we investigated the effects of activated SGCs on synapse formation in vitro. Immunofluorescence staining of synapsin and PSD95 was used to label the synapses in cultured neurons (Fig. 6D). When primary neurons were cultured in SGC-conditioned medium for 24 h, the synapse number significantly decreased (Control vs 0.5 μg/ml: 1.0 ± 0.1 fold vs 0.4 ± 0.2 fold, p = 0.002; Control vs 2.0 μg/ml: 1.0 ± 0.1 fold vs 0.1 ± 0.07 fold, p < 0.001; Control vs LPS: 1.0 ± 0.1 fold vs 0.4 ± 0.2 fold, p = 0.004) (Fig. 6E). Together, these findings suggest that activated SGCs are neurotoxic and have detrimental effects on synapse formation and maintenance.

### Interactions between activated SGCs and macrophages occurred around the primary sensory neurons in the DRG of L5 NRL rats

**Fig. 7:**
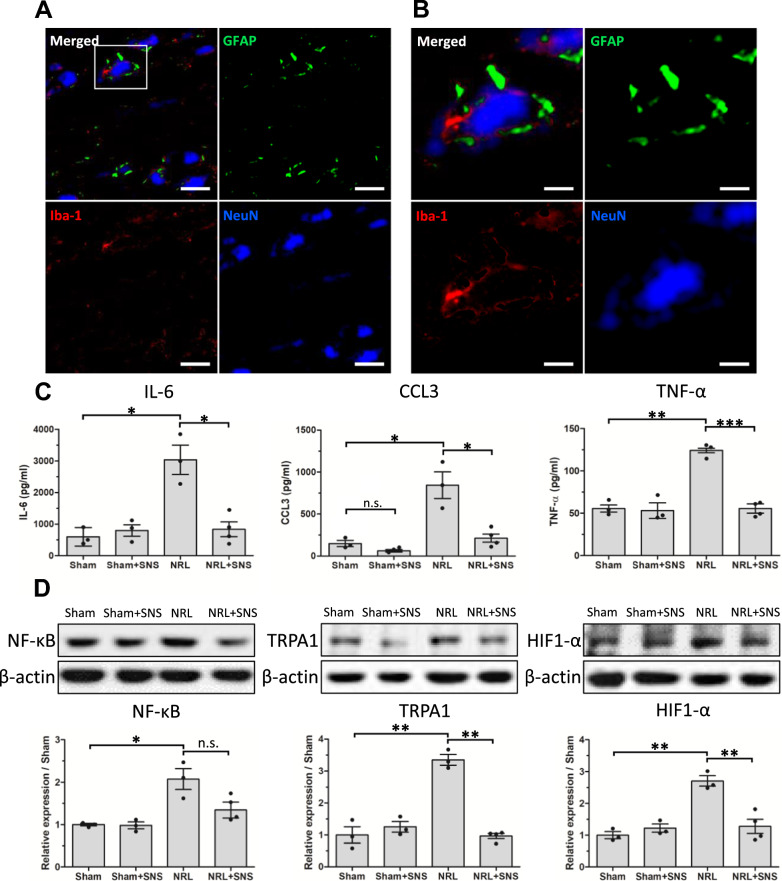
Interactions between activated SGCs and macrophages via cytokines and upregulation of HIF-1α and NF-κB pathways. **A** Representative confocal microscopy images of glial fibrillary acidic protein (GFAP), ionized calcium binding adaptor molecule 1 (Iba-1), and NeuN in the L5 DRG following nerve root ligation. The magnified region is indicated by the white rectangle. Scale bars = 50 μm. **B** Magnified confocal microscopy images of GFAP, Iba-1, and NeuN showing nearby signals of GFAP and Iba-1 surrounding NeuN signals. Scale bars = 10 μm. **C** Enzyme-linked immunosorbent assay quantifications of interleukin-6, chemokine (C–C motif) ligand 3, and tumor necrosis factor-α in the L5 DRG on post-injury day (PID) 8 (N = 3). **D** Representative Western blots of NF-κB, TRPA1, and HIF1-α in the L5 DRG on PID 8. The corresponding quantifications of relative protein levels for NF-κB, TRPA1, and HIF1- α are also shown (N = 3). Data are expressed as mean ± standard deviation. *p < 0.05, **p < 0.01, ***p < 0.01

Previous studies have shown that neurotoxic astrocytes are induced by activated microglia in the central nervous system (CNS) [23–25]. Therefore, in analogy, we investigated whether macrophages interact with SGCs. To investigate the interactions between DRG macrophages and SGCs, we performed immunofluorescence staining for NeuN, GFAP, and ionized calcium binding adaptor molecule 1 (Iba-1) in the DRG of NRL rats in PID 8. Triple staining showed the nearby signals of the activated SGC marker GFAP and the activated macrophage marker Iba-1 (Fig. 7A). Additionally, in the magnified images (Fig. 7B), the nearby GFAP and Iba-1 signals encircled the NeuN signal, indicating that primary afferent neurons were surrounded by activated SGCs and macrophages.

Reciprocal activation between SGCs and macrophages relies on the paracrine secretion of inflammatory cytokines [26, 27]. Therefore, we performed enzyme-linked immunosorbent assay (ELISA) analysis to quantify the levels of IL-6, chemokine (C–C motif) ligand 3 (CCL3), and TNF-α in the DRG on PID 8 (Fig. 7C). Compared with Sham rats, NRL rats had significantly elevated levels of IL-6 (599.6 ± 410.9 pg/ml vs 3037.4 ± 803.9 pg/ml, p = 0.016), CCL3 (149.86 ± 45.2 pg/ml vs 844.0 ± 173.6 pg/ml, p = 0.042), and TNF-α (55.5 ± 5.9 pg/ml vs 124.2 ± 3.8 pg/ml, p = 0.009). Additionally, the application of SNS reduced the levels of IL-6 (3037.4 ± 803.9 pg/ml vs 629.4 ± 257.0 pg/ml, p = 0.024), CCL3 (844.0 ± 173.6 pg/ml vs 184.2 ± 64.5 pg/ml, p = 0.047), and TNF-α (124.2 ± 3.8 pg/ml vs 52.5 ± 11.0 pg/ml p < 0.001) induced by L5 NRL. Analysis of the mRNA levels yielded similar results, showing that SNS application alleviated the elevated mRNA levels of inflammatory cytokines induced by NRL (Supplementary Figure S6).

Further, Western blot analyses showed increased protein expressions of NF-κB (1.0 ± 0.1 fold vs 2.1 ± 0.4 fold, p = 0.047), hypoxia inducible factor 1 subunit alpha (HIF-1α) (1.0 ± 0.2 fold vs 2.7 ± 0.3 fold, p = 0.002), and TRPA1 (1.0 ± 0.4 fold vs 3.3 ± 0.3 fold, p = 0.003) (Fig. 7D), as well as elevated mRNA levels (Supplementary Figure S7) following NRL, and the elevated expressions of HIF-1α (2.7 ± 0.3 fold vs 1.4 ± 0.4 fold, p = 0.004) and TRPA1 (3.3 ± 0.3 fold vs 1.0 ± 0.1 fold, p = 0.001) were alleviated by SNS. The reduction of NF-κB expression in the NRL + SNS group was borderline significant compared with the NRL group (2.1 ± 0.4 fold vs 1.4 ± 0.3 fold, p = 0.082).

Together, these results demonstrate the spatial interaction between activated macrophages and SGCs occurring around primary sensory neurons in the DRG, and the upregulation of pathways involved in glial activation and NP [28, 29].

### Effects of SNS on macrophage polarization in the DRG in L5 NRL rats

Based on our finding that L5 NRL and SNS modulated the activation of DRG SGCs, and the interactions between SGCs and macrophages, we next investigated whether NRL and SNS altered the activation and polarization of DRG macrophages.

**Fig. 8:**
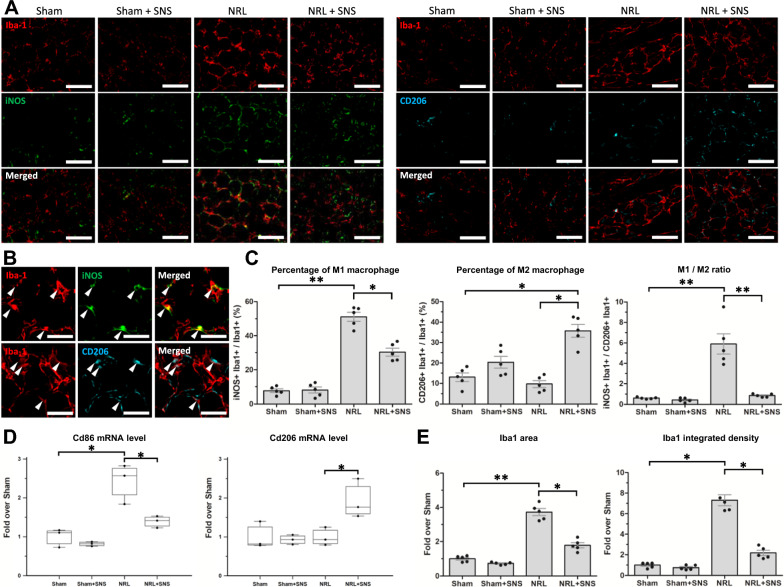
NRL and SNS altered the macrophage polarization in the L5 DRG. **A** Immunofluorescence staining of satellite glial cell macrophage marker ionized calcium binding adaptor molecule 1 (Iba-1) (red), M1 marker inducible nitric oxide synthase (green) and M2 marker CD206 (cyan) on post-injury day (PID) 8 (N = 5). **B** Representative magnified images of M1 and M2 macrophages indicated by arrowheads. **C** The percentages of M1 and M2 macrophages and the ratio of M1/M2 quantified using ImageJ software. **D** The messenger RNA levels of Cd86 and Cd206 in the L5 DRG on PID 8 quantified by NanoString nCounter. **E** The relative area and integrated density of the Iba-1 signal quantified using ImageJ software. Data are expressed as mean ± standard deviation. Scale bars = 200 μm and 50 μm. *p < 0.05, **p < 0.01

Immunofluorescence staining of the M1 and M2 macrophage markers, inducible nitric oxide synthase (iNOS) and CD206, was performed (Fig. 8A, B). Quantification of iNOS + /Iba-1 + and CD206 + /Iba1 + cells on PID 8 showed that compared with the Sham group, the NRL group had an increased M1 percentage (7.8 ± 2.3% vs 51.1 ± 5.9%, p = 0.005) and M1/M2 ratio (0.6 ± 0.1 vs 5.9 ± 2.2, p = 0.008), whereas compared with the NRL group, the NRL + SNS group had a decreased M1 percentage (51.1 ± 5.9% vs 30.3 ± 5.1%, p = 0.019) and M1/M2 ratio (5.9 ± 2.2% vs 0.9 ± 0.2, p = 0.009) and an increased M2 percentage (9.7 ± 3.8% vs 35.7 ± 7.0%, p = 0.027) (Fig. 8C). Likewise, quantification of Cd86 and Cd206 mRNA expression levels showed that NRL induced an increase in Cd86 mRNA expression (1.0 ± 0.2 fold vs 2.3 ± 0.6 fold, p = 0.028), whereas NRL + SNS decreased Cd86 (2.3 ± 0.6 fold vs 1.4 ± 0.2 fold, p = 0.043) and increased Cd163 mRNA expression (1.1 ± 0.1 fold vs 1.9 ± 0.5 fold, p = 0.049) compared with NRL (Fig. 7D). Quantification of Iba-1 signals showed an increased Iba-1 area (1.0 ± 0.2 fold vs 3.7 ± 0.5 fold, p = 0.009) and integrated density (1.0 ± 0.3 fold vs 7.3 ± 1.2 fold, p = 0.019) following NRL, both of which were reduced in the NRL + SNS group (area: 3.7 ± 0.5 fold vs 1.8 ± 0.3, p = 0.011; density: 7.3 ± 1.2 fold vs 2.2 ± 0.6 fold, p = 0.020) (Fig. 8E). These results demonstrate that L5 NRL induces a shift in DRG macrophage polarization toward an M1-predominant phenotype, whereas the application of SNS induces an increase in M2 polarization.

## Discussion

Detailed characterization of the interplay between DRG SGCs, macrophages, and primary sensory neurons in a model of NRL and SNS will advance our understanding of the pathobiology of NP and improve the application of SNS neuromodulation. This study shows that DRG SGCs play a pivotal role in the pathogenesis of NP following NRL, which induced activation of SGCs, marked by increased TPRA1, Cx43, and gap junctions regulated by the HIF-1α-NF-κB pathways, amplifying the pain-related signal transmission. Moreover, reciprocal activation between activated SGCs and M1 macrophages involved spatial interactions and the release of inflammatory cytokines, including IL-6, CCL3, and TNF-α in the DRG and surrounding primary sensory neurons. Furthermore, we unveiled the modulatory effect of SNS, which decreased SGC activation and increased M2 macrophage polarization.

In a previous study, we demonstrated the therapeutic potential of 20-Hz SNS in alleviating NP and spinal cord neuroinflammation [10]. However, in our previous study, SNS was performed 2 h after NRL, making it less clinically applicable [10]. Therefore, in this study, we performed SNS on PID 1 to broaden its applicability for future clinical translation. Our behavioral experiments showed that neuromodulation with the SNS performed on PID 1 effectively alleviated NP behaviors, including mechanical allodynia and thermal hypersensitivity, from PIDs 2 to 15. Furthermore, since the effect of SNS on the DRG remains unclear, the present study focused on the neural-glial-macrophage interactions that occurred in the DRG. As the gateway for peripheral-to-central pain transmission, the DRG is upstream of the neuroinflammatory cascade initiated by nerve injury, spreading from the peripheral nerves to the spinal cord [13, 14, 30, 31]. Guan et al. found that inflammatory mediators, such as CSF1, are transported from injured primary sensory neurons to the spinal cord, triggering subsequent neuroinflammation in the CNS [14]. Therefore, a detailed investigation of the cellular interactions within the DRG could enhance the application of SNS and benefit patients with NP. Our study provides valuable insight into these issues.

First, we investigated alterations in gene expression in the DRG after NRL and SNS. Using NanoString nCounter technology, we simultaneously analyzed the expression levels of 770 signature genes involved in neuroinflammation, this powerful tool enables the detection of potentially differentially expressed molecular and cellular pathways. Upon reviewing the gene analysis, the significant alterations in the glial cell pathway drew our attention. In DRG, SGCs are the primary glial cells that maintain neuronal microenvironment homeostasis via the regulation of glutamate and potassium concentrations and respond to injury with gliosis and expression of GFAP [31–33]. Our results demonstrated that SGCs were hyperactivated after NRL. Furthermore, the application of SNS to NRL rats not only alleviated pain behaviors but also decreased SGC activation, marked by a significantly reduced GFAP signal. Taken together, these results suggest that SGC played a critical role in L5 NRL-induced NP and SNS exerts a modulatory effect on SGC following NRL, which might contribute to alleviation of NP.

Second, to elucidate the mechanisms by which activated SGCs contribute to NRL-induced NP, we investigated the molecular and cellular events occurring in SGCs following NRL. The abnormal expression of ion channels in DRG SGC plays an essential role in the initiation and maintenance of NP after nerve injury [18–20]. Among these channels, TRPA1 is a transient receptor potential cation channel that is expressed in both primary sensory neurons and SGCs [19]. Axonal injury and oxidative stress induce increased membrane expression of TRPA1, resulting in calcium influx, hyperexcitation of neurons and SGCs, and disruption of glutamatergic homeostasis, all contributing to NP sensitization and maintenance [19, 34]. Our study revealed that NRL-induced TPRA1 expression was mainly localized to SGCs rather than primary sensory neurons, indicating a central role of gliopathy in NRL-induced pain. Further, SGCs also exhibit the ability to couple via gap junctions. The presence of SGC–SGC and SGC–neuron coupling could further explain how SGC hyperexcitation spreads to sensory neurons [35]. Cx43 is a major component of gap junctions in glial cells [36–38]. Tonkin et al. showed that elevated Cx43 expression and increased SGC coupling are associated with excessive glial activation and pain [36]. Moreover, an increased number of gap junctions can facilitate the paracrine secretion of proinflammatory cytokines, leading to NP [37].

Third, in the nCounter and western blot analyses, we observed elevated mRNA and protein expression of HIF-1α and NF-κB in the DRG following NRL, respectively. Alterations in the expressions of TRPA1 and Cx43 can be regulated by the HIF-1α-NF-κB pathway [28, 39]. Hatano et al. reported that HIF-1α could induce increased expression of TRPA1 through regulation of NF-κB, thereby permitting calcium inflow and contributing to hyperexcitation of SGCs [28]. Excessive opening of Cx43 hemichannels was reported to exacerbate the inflammatory response with upregulation of NF-Κb [28]. Moreover, increased gap junction plaques between the GCSs and neurons in the DRG visualized by TEM could facilitate the transportation of calcium from activated SGCs to the encircled primary sensory neurons, thereby contributing to neuronal hyperexcitation and NP. Together, these molecular events offered mechanistic explanations of how alterations in SGC function contribute to NRL-induced NP development. Moreover, SNS could alleviate these pathological alterations in the SGCs, marked by reduced TRPA1, Cx43, HIF-1α, NF-κB, and gap junctions after SNS. Collectively, these results revealed that following NRL, both the molecular markers and functions of SGCs undergo alteration, and these changes could be reversed by SNS.

Finally, the interplay between SGCs, macrophages, and primary sensory neurons in the DRG was analyzed. Macrophages in the DRG are required for the initiation and maintenance of NP [40, 41]. Specifically, M1 macrophages infiltrate the DRGs after nerve injury and communicate with SGCs and sensory neurons via cytokines, leading to nociceptive excitability and prolonged sensitization [15, 27]. Inflammatory cytokines are pivotal mediators of the interactions between macrophages and SGCs [40]. CCL3 secreted from injured sensory neurons attracts macrophages to the DRG and promotes M1 polarization [23, 42]. Moreover, activated SGCs release IL-6 and TNFα, which act on the neurons and macrophages, resulting in increased excitability and M1 polarization, respectively [2]. It is reasonable that CCL3, IL-6, and TNFα were involved in the interactions between the sensory neurons, M1 macrophages, and activated SGCs, contributing to the NP following NRL [23, 42]. Importantly, we also observed spatial interactions between macrophages, SGCs, and sensory neurons in the DRG. The nearby Iba-1 and GFAP signals suggest proximity between macrophages and SGCs and could facilitate paracrine communication via inflammatory cytokines [27, 43]. These results imply that reciprocal activation between M1 macrophages and activated SGCs around the sensory neurons in the DRG contributes to the formation and maintenance of NRL-induced NP. Notably, SNS was able to alleviate SGC activation, shift M1 macrophage to M2, and decrease the elevated expression of inflammatory cytokines.

Collectively, our results suggest that L5 NRL induces the activation of DRG SGCs into an inflammatory and neurotoxic phenotype. Activated SGCs exhibit increased expression of ion channels that contributed to the amplification of pain-related signal transmission and engaged in bidirectional communication with activated M1 macrophages via spatial interactions and the release of inflammatory cytokines surrounding the primary sensory neurons. Mechanistically, the upregulated HIF-1α-NF-κB pathway explained the activation of SGCs and macrophages. Neuromodulation with SNS effectively reversed SGC activation, reduced M1 macrophage polarization, mitigated inflammatory phenotypes, and alleviated NP.

In additions, we evaluated the effect of SNS on healthy subjects, specifically present as the Sham + SNS group, in which the L5 nerve root was not ligated and SNS was performed. Our findings showed that compared to the Sham group, rats in the Sham + SNS group did not have de novo pain behaviors and DRG neurons did not showed increased ATF3 expression. Moreover, nCounter analysis did not show significant differences in the neuroinflammation pathway scores and immunofluorescence of DRG SGC and macrophages did not show significant changes in the Sham + SNS group compared to Sham group. Therefore, these results provided insights in the safety of SNS, which could facilitate future clinical translation for patients suffering from NP.

While our study demonstrated the efficacy of SNS in the modulation of DRG SGCs and explored relevant molecular pathways, the exact bioelectric mechanism of electrical peripheral neurostimulation remained unexplored. Future studies should investigate the intracellular and intercellular events within DRG cells following electrical modulation. The specific cell-type pathways underlying NRL-induced NP remain unknown. Further in vitro studies using isolated macrophages, SGC, and sensory neurons may help identify these pathways. Nonetheless, our results showed a significant elevation of the HIF-1α and NF-κB pathways following NRL in the DRG, providing valuable directions for future investigations. Understanding the basis of these processes can provide insights into improving currently available neuromodulation techniques for the benefit of patients with NP.

## Conclusions

SGC played a pivotal role in NP. NRL induced hyperactivation of DRG SGCs, which had increased expression of TRPA1 and Cx43 ion channels that contributed to NP. Reciprocal activation of SGCs and macrophages surrounding the primary sensory neurons was mediated by spatial interactions and inflammatory cytokines, via the HIF-1α and NF-κB pathways. The gliopathic alterations and the pain behaviors were mitigated by SNS, which had a modulatory effect that suppressed SGC hyperactivation and skewed M1 macrophage polarization towards M2. Our findings establish SGC activation as a crucial pathomechanism in the gliopathic alterations associated with NRL-induced NP, which can be modulated by SNS neuromodulation.

### Supplementary Information

Supplementary material 1.

## Data Availability

The datasets used and/or analysed during the current study are available from the corresponding author on reasonable request.

## Abbreviations

CCL3: Chemokine (C–C motif) ligand 3
CNS: Central nervous system
CSF1: Colony-stimulating factor 1
Cx43: Connexin 43
DEG: Differentially expressed gene
DIV: Days in vitro
DRG: Dorsal root ganglia
ELISA: Enzyme-linked immunosorbent assay
GFAP: Glial fibrillary acidic protein
IL: Interleukin
LPS: Lipopolysaccharide
NF-κB: Nuclear factor kappa-light-chain-enhancer of activated B cells
NP: Neuropathic pain
NRL: Nerve root ligation
SGC: Satellite glial cell
SNS: Sciatic nerve stimulation
TNF: Tumor necrosis factor
TRPA1: Transient receptor potential cation channel subfamily A member 1
