# Supplementary material for: Sciatic nerve stimulation alleviates neuropathic pain and associated neuroinflammation in the dorsal root ganglia in a rodent model
Source: J Transl Med. 2024 Aug 14;22:770. doi: 10.1186/s12967-024-05573-1 (PMC11325705; doi:10.1186/s12967-024-05573-1)
Supplement: Supplementary file 1 — Supplementary material 1. [file 12967_2024_5573_MOESM1_ESM.pdf]

**Supplementary table 1** Differentially expressed genes between experimental groups

| Differentially expressed genes between groups: Sham v.s. Sham+SNS |
|---|
| Anxa1, Apex1, Arid1a, Atg5, Atp6v0e, Bad, Bard1, Bbc3, Bcl2, Bcl2l1, Becn1, Birc2, Birc5, Blnk, Braf, Brca1, Brd2, Casp2, Casp3, Casp4, Ccni, Cd47, Chek1, Chek2, Chuk, Creb1, Crebbp, Cryba4, Csk, Ctse, E2f1, Eed, Ehmt2, Entpd2, Ep300, Ezh2, Fancc, Fancd2, Gclc, Gria4, Hat1, Hdac1, Hells, Hira, Igf1r, Ikbkb, Ikbkg, Irak1, Itgb5, Kdm1a, Kdm2a, Kdm2b, Kdm5a, Kdm5d, Kdm6a, Kmt2a, Lamp2, Lfng, Lig1, Map3k1, Mapk14, Mbd2, Mcm2, Mcm6, Mfge8, Msh2, Myc, Nbn, Ncaph, Ncor1, Nfkb1, Nfkbia, Nfkbie, Nlgn2, Nrp2, Parp2, Pcna, Pik3cg, Plcg2, Pld1, Plekhm1, Plp1, Pole, Pten, Ptms, Ptpn6, Pttg1, Rad1, Rad51, Rad9a, Rala, Rhoa, Rpa1, Rps9, Rrm2, S1pr4, Setd1b, Setd2, Sin3a, Smarca4, Smarca5, Spint1, Steap4, Stmn1, Stx18, Suv39h1, Suz12, Tarbp2, Timp1, Tmem206, Top2a, Tradd, Traf6, Uty, Vps4b, Wdr5, Xiap |
| **Differentially expressed genes between groups: Sham v.s. NRL** |
| Abl1, Akt1, Ambra1, Anapc15, Anxa1, Apc, Apex1, Apoe, Arid1a, Atf3, Atg14, Atg3, Atg5, Atg7, Atg9a, Atp6v0e, Atp6v1a, Bad, Bag3, Bak1, Bbc3, Bcl2, Bcl2l1, Becn1, Bin1, Birc2, Bola2, Braf, Brd2, Brd4, C1qa, C1qb, C1qc, C3, C3ar1, Cables1, Calr, Casp2, Casp3, Casp4, Casp6, Ccl2, Ccl5, Ccni, Cd3e, Cd47, Cd68, Cd74, Cdk20, Cflar, Chek1, Chek2, Chuk, Cks1b, Col6a3, Cotl1, Cox5b, Cp, Creb1, Crebbp, Crip1, Csf1, Csk, Ctsf, Ctss, Cyp27a1, Cyp7b1, Dab2, Ddx58, Dicer1, Dlg1, Dlg4, Dock1, Dock2, Dot1l, Dst, Eed, Eef2k, Egfr, Ehmt2, Entpd2, Ep300, Epg5, Erbb3, Ezh1, Ezh2, Fbln5, Fcer1g, Fgd2, Fos, Fscn1, Gadd45a, Gadd45g, Gja1, Gna15, Grap, Gria1, Gria4, Grn, Hat1, Hdac1, Hdac4, Hdac6, Hif1a, Hira, Hmox1, Homer1, Hspb1, Ifih1, Ifitm2, Igf1, Igf1r, Igf2r, Ikbkb, Ikbkg, Il10rb, il1a, Il1b, Il1rap, Il1rl2, Il1rn, Il2rg, Il6, Inpp5d, Irak1, Irak2, Irf1, Irf2, Itga7, Itgb5, Jag1, Jarid2, Jun, Kat2a, Kat2b, Kcnj10, Kdm1a, Kdm2a, Kdm2b, Kdm3a, Kdm4c, Kdm5a, Kdm5c, Kdm5d, Kdm6a, Kmt2a, Lacc1, Lamp2, Lfng, Lgmn, Lilrb4a, Lmna, Map1lc3a, Map2k1, Map2k4, Map3k1, Mapk14, Mbd2, Mbd3, Mdm2, Mertk, Mfge8, Mmp12, Mmp14, Msh2, Myc, Nbn, Ncf1, Ncor1, Ncor2, Nfkb1, Nfkb2, Nfkbia, Nfkbie, Ngfr, Nlgn1, Nlgn2, Nlrp3, Npl, Nrm, Nrp2, Nthl1, Olfml3, Optn, P2rx7, P2ry12, Pak1, Parp2, Pcna, Pex14, Pik3ca, Pik3cb, Pik3cg, Pik3r1, Pik3r2, Pla2g4a, Pld1, Pld2, Plekhm1, Plp1, Plxdc2, Plxnb3, Pms2, Ppp3ca, Ppp3cb, Ppp3r1, Prkaca, Prkdc, Psen2, Psmb8, Pten, Ptms, Rab7, Rad1, Rad51, Rad9a, Rala, Rb1cc1, Rela, Relb, Reln, Rhoa, Ripk2, Rpa1, Rpl28, Rpl29, Rpl36al, Rpl9, Rps10, Rps2, Rps21, Rps9, S100a10, S1pr4, Serping1, Sesn1, Sesn2, Setd1a, Setd1b, Setd2, Setd7, Shank3, Sin3a, Sirt1, slc1a3, Smarca4, Smarca5, Smc1a, Socs3, Sox10, Sox4, Stat1, Stmn1, Stx18, Suv39h1, Suz12, Tarbp2, Tcirg1, Tgfb1, Tgfbr1, Tgm1, Tgm2, Tie1, Timp1, Tlr7, Tm4sf1, Tmc7, Tmem206, Tmem64, tnfa, Tnfrsf12a, Tradd, Traf2, Traf3, Traf6, Trem2, Trp53bp2, Trpm4, Tspan18, Ulk1, Ung, Uty, Vav1, Vegfa, Vim, Vps4a, Vps4b, Wdr5, Xiap, Zfp367 |
| **Differentially expressed genes between groups: Sham v.s. NRL+SNS** |
| Apoe, Atg14, Atg5, Atg7, Bcl2l11, C1qa, C1qc, C3, C3ar1, Casp3, Casp4, Casp6, Cd3e, Cd68, Cd74, Cflar, Chek2, Col6a3, Cotl1, Cp, Crip1, Ctss, Cyp7b1, Dab2, Ddx58, Dock1, Ehmt2, Entpd2, Erbb3, Ezh2, Fcer1g, Gna15, Gria4, Hdac4, Hif1a, Ifih1, Ifitm2, Igf1, Il10rb, Il1rap, Irak2, Irak4, Irf1, Itga7, Jun, Kdm2b, Kdm3a, Lamp2, Lfng, Lgmn, Lilrb4a, Mdm2, Mertk, Mmp12, Ncf1, Nfkb1, Nfkb2, Nlrp3, Npl, Olfml3, P2rx7, P2ry12, Pik3cg, Pld1, Pld2, Plxdc2, Prkdc, Psmb8, Pttg1, S1pr4, Sesn2, Snca, Socs3, Sox4, Tgfbr1, Tie1, Timp1, Tlr7, Tmc7, Tnfrsf12a, Trem2, Ung, Uty, Vav1 |

| Differentially expressed genes between groups: NRL v.s. NRL+SNS |
|---|
| Abl1, Adamts16, Akt1, Ambra1, Anapc15, Apc, Apex1, Arid1a, Atf3, Atg3, Atg5, Atg7, Atg9a, Atp6v0e, Atp6v1a, Atr, Bad, Bag3, Bard1, Bax, Bcl2, Bcl2l1, Becn1, Bola2, Braf, Brd2, Brd4, C1qb, C3, Cables1, Calr, Casp3, Casp4, Ccl2, Ccl3, Ccni, Cd163, Cd47, Cd68, Cdk20, Cflar, Chek1, Chuk, Clstn1, Cntnap2, Cox5b, Creb1, Crebbp, Cryba4, Csf1, Csk, Ctsf, Dicer1, Dlg4, Dot1l, Dst, Eed, Ep300, Epg5, Ezh1, Ezh2, Fos, Fscn1, Gadd45a, Gadd45g, Gclc, Gja1, Gria1, Gria2, Gria4, Hat1, Hdac1, Hdac6, Hif1a, Hira, Homer1, Hspb1, Ikbkb, Ikbkg, Il10rb, Il1b, Il6, Irak1, Itga7, Jag1, Jarid2, Jun, Kat2a, Kdm1a, Kdm2a, Kdm3a, Kdm4c, Kdm5c, Kdm5d, Kdm6a, Kmt2a, Lamp2, Lingo1, Map2k1, Map2k4, Mapk12, Mapk14, Mapt, Mbd2, Mdm2, Nbn, Ncor1, Ncor2, Nfkb1, Nfkbia, Nlgn1, Nlgn2, Nqo1, Optn, Pak1, Pcna, Pex14, Pik3ca, Pik3cb, Pik3r1, Pik3r2, Pld2, Plekhm1, Pms2, Ppfia4, Ppp3ca, Ppp3cb, Ppp3r1, Prkaca, Prkar2b, Prkce, Prkdc, Psen2, Pten, Ptms, Rab7, Rala, Rb1cc1, Rhoa, Ripk2, Rpa1, Rpl36al, Rps21, S100a10, Sesn1, Sesn2, Setd1a, Setd1b, Setd2, Setd7, Sin3a, slc1a3, Smarca4, Smarca5, Smc1a, Socs3, Stmn1, Suz12, Tarbp2, Timp1, Tm4sf1, Tmem206, Tnfrsf12a, Traf6, Trp53bp2, Trpa1, Trpm4, Tspan18, Ulk1, Ung, Uty, Vegfa, Vps4a, Vps4b, Wdr5, Xiap, Zfp367 |

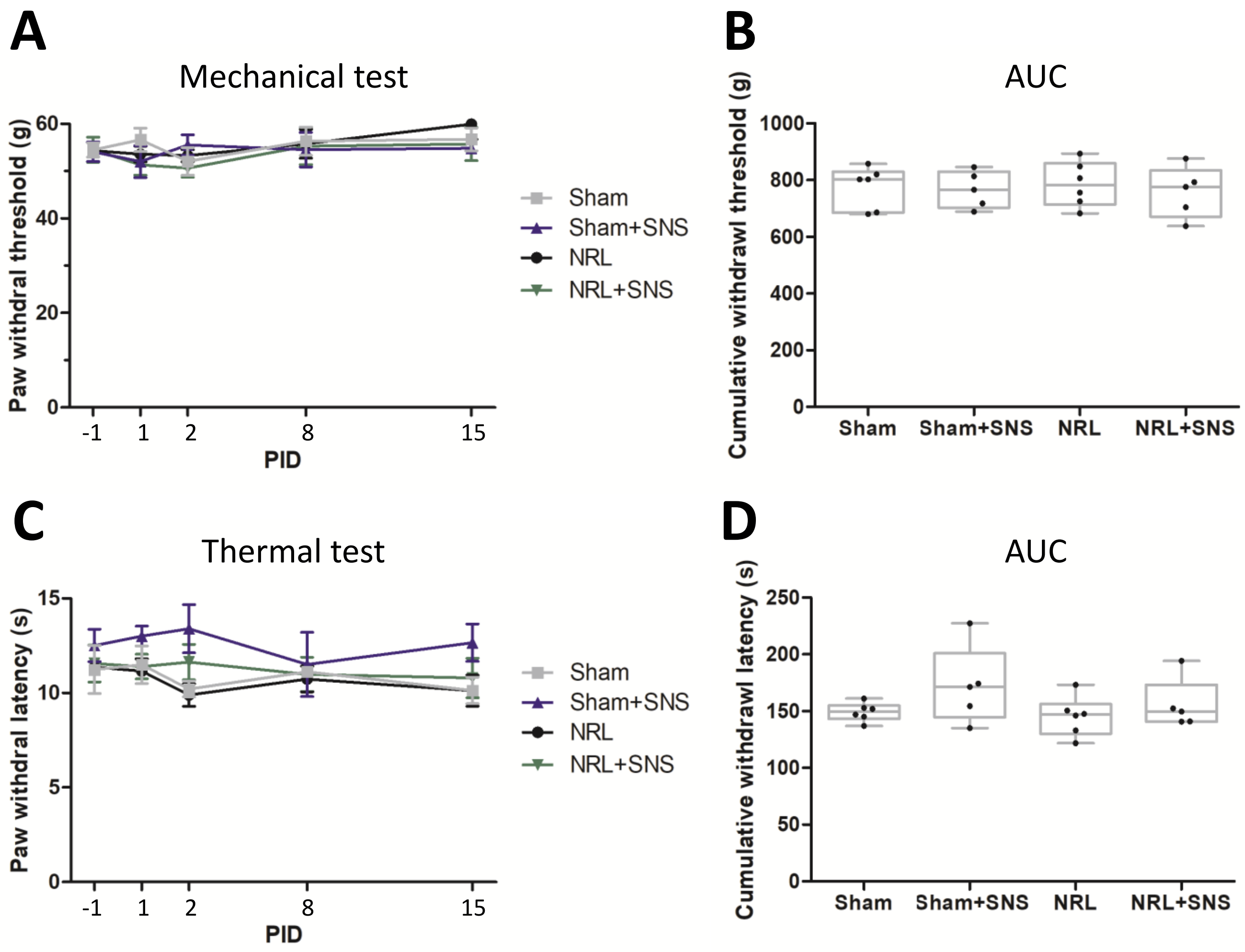


**Supplementary figure S1** Contralateral mechanal and thermal pain behavioral results.

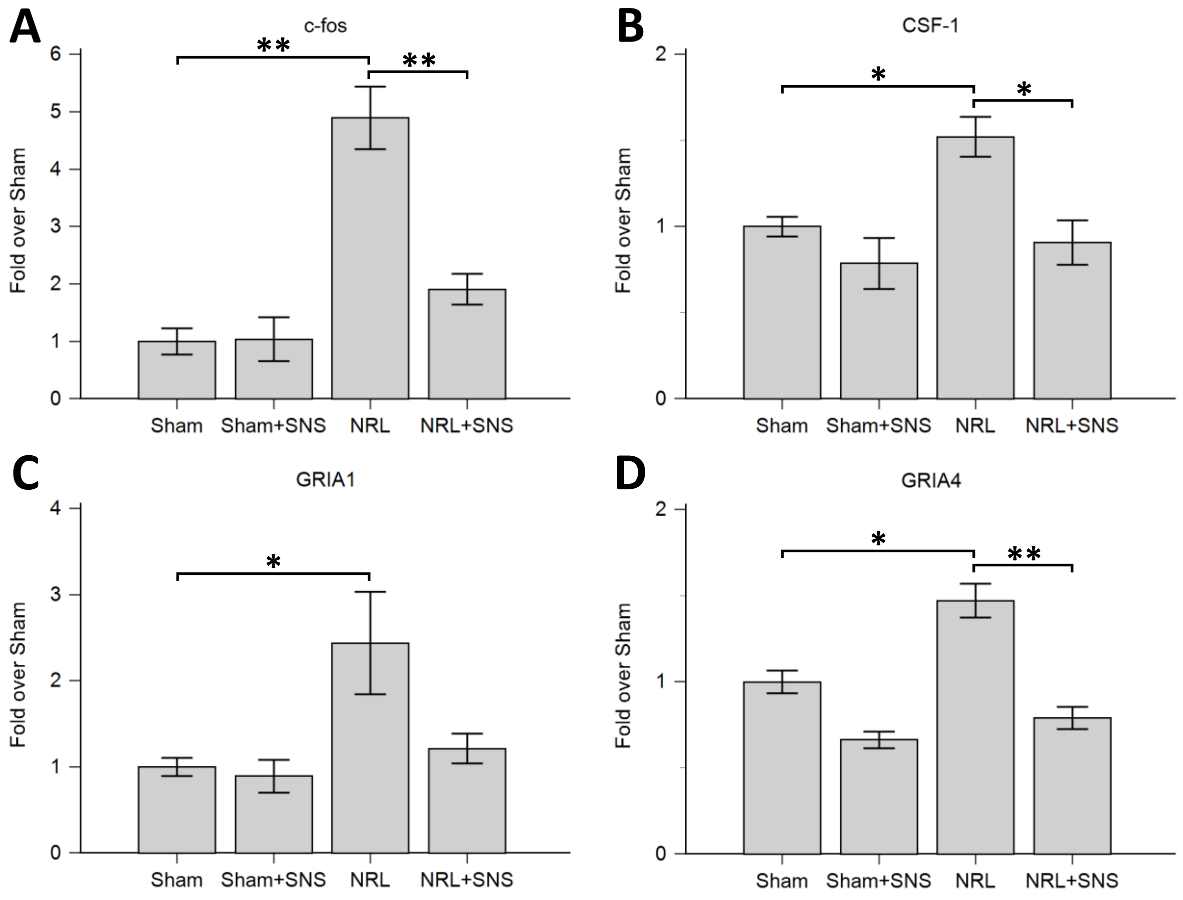


**Supplementary figure S2** The mRNA levels of genes associated with injury of primary afferent neurons and neuropathic pain quantified by nanostring nCounter assay.

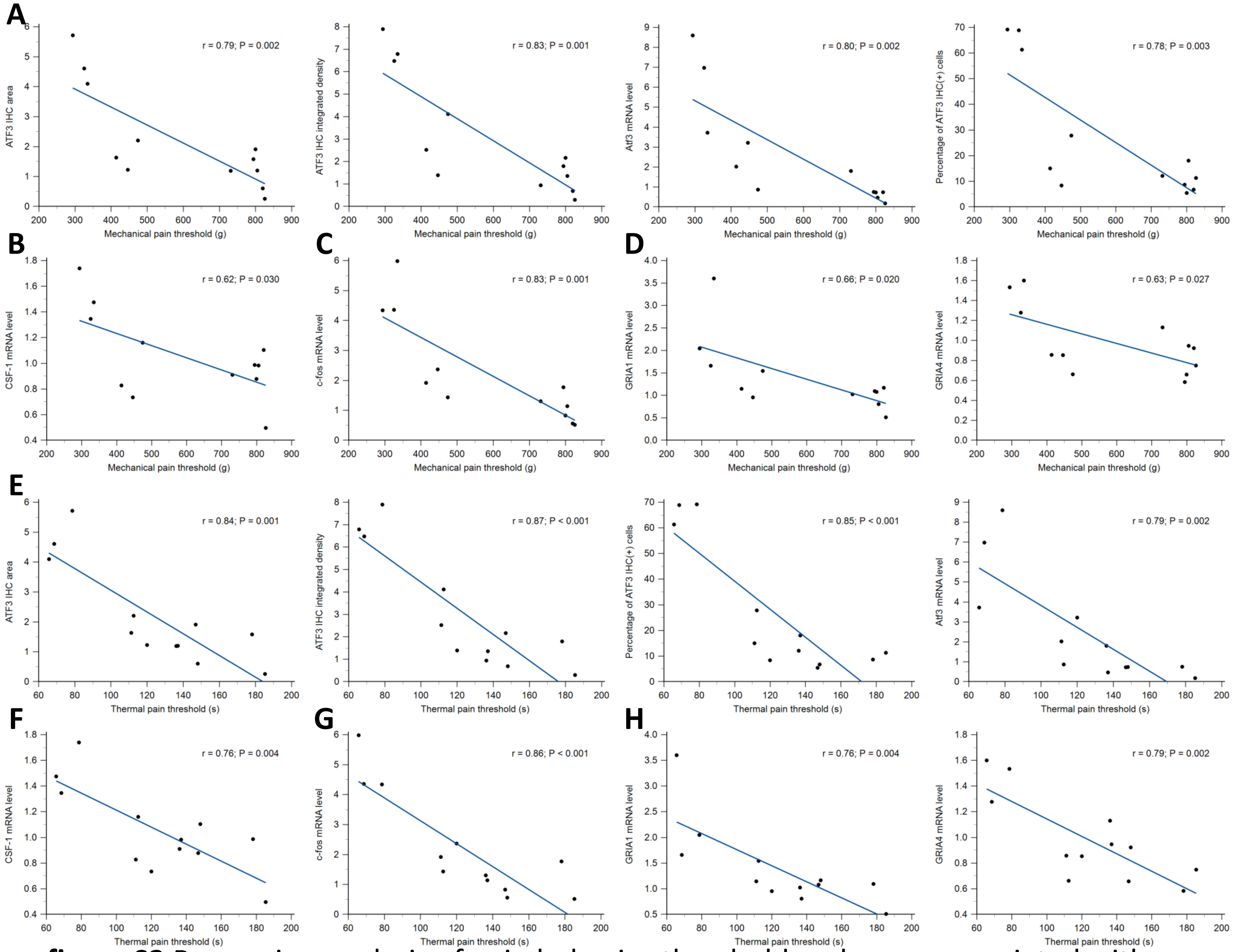


**Supplementary figure S3** Regression analysis of pain behavior threshold and genes associated with neuropathic pain.

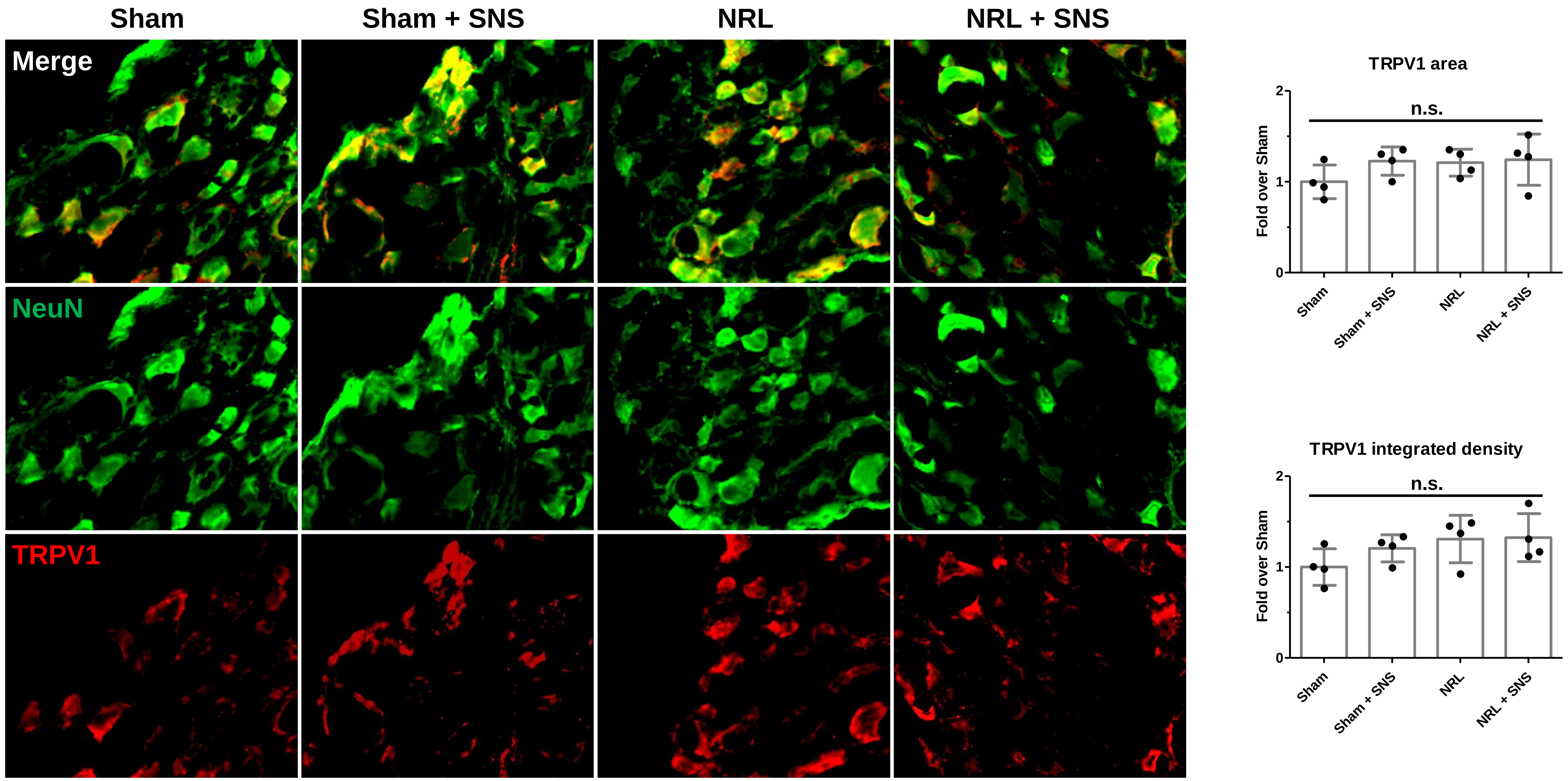


**Supplementary figure S4** Immunoflurescence of TRPV1 and NeuN in DRG

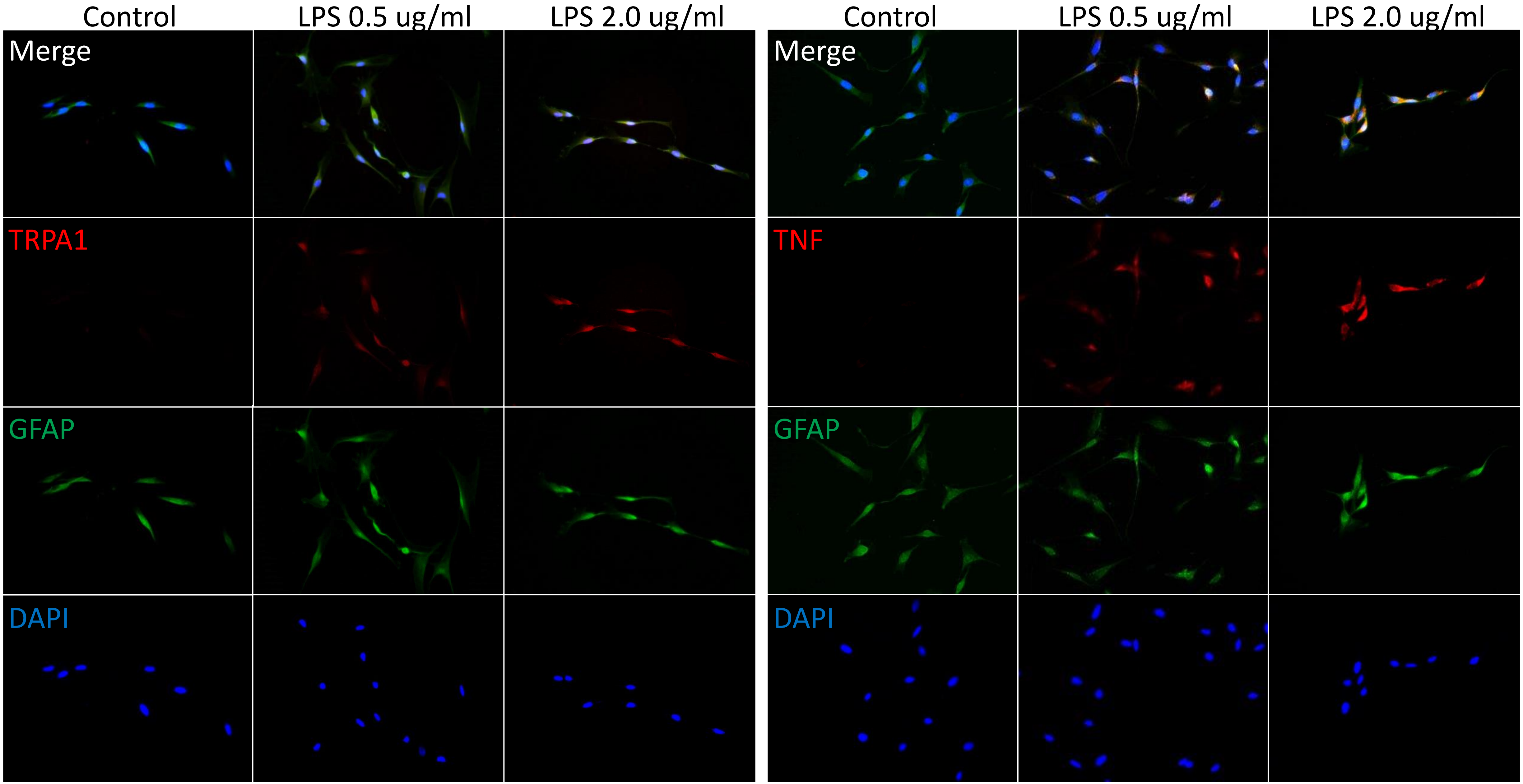


**Supplementary figure S5** Immunoflurescence of TRPA1, TNF, and GFAP in cultured SGCs treated with LPS

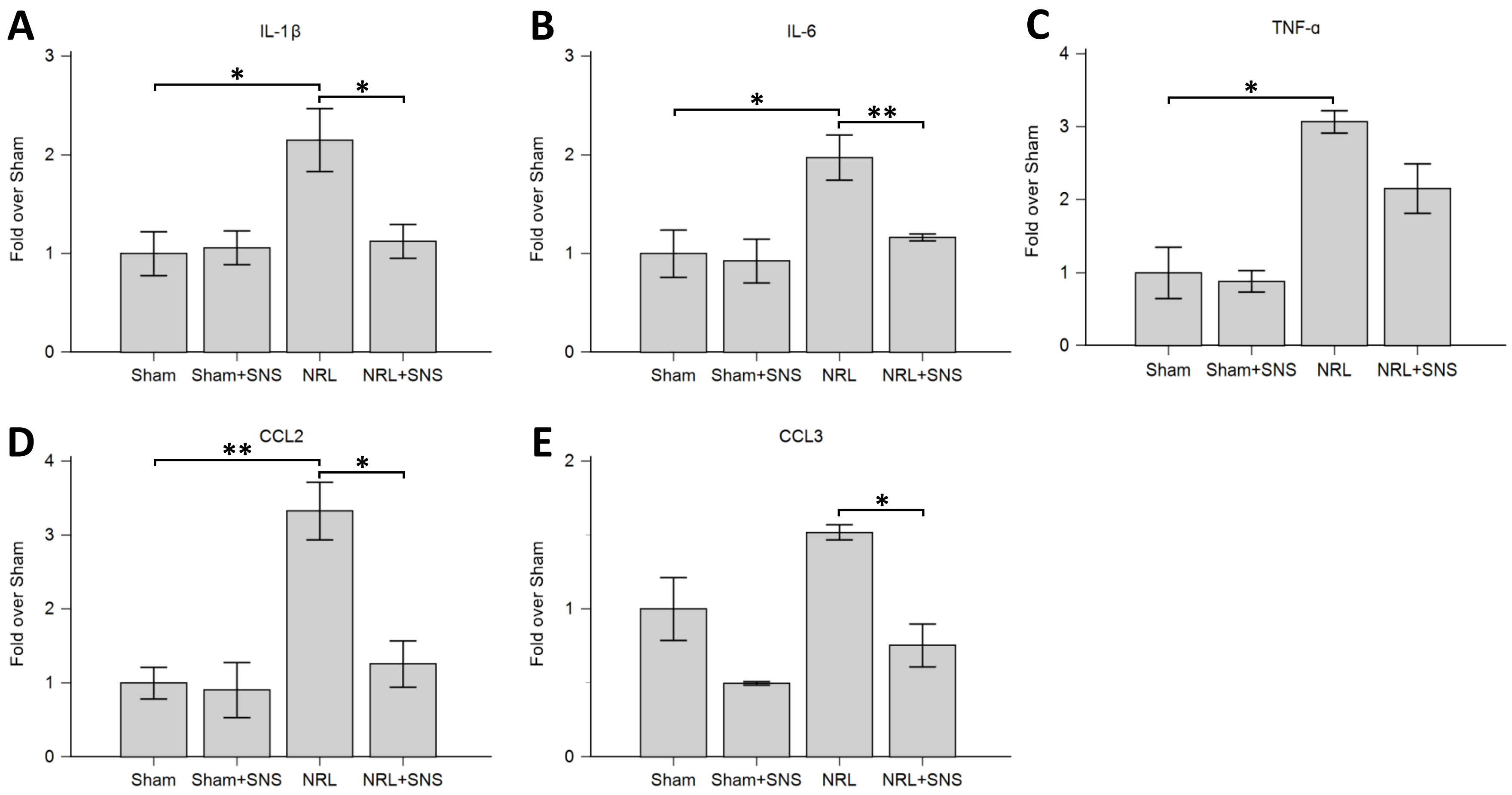


**Supplementary figure S6** The mRNA levels of cytokines involved in the interactions between the infiltrating macrophages and satellite glial cells in the DRG quantified by nanostring nCounter assay.

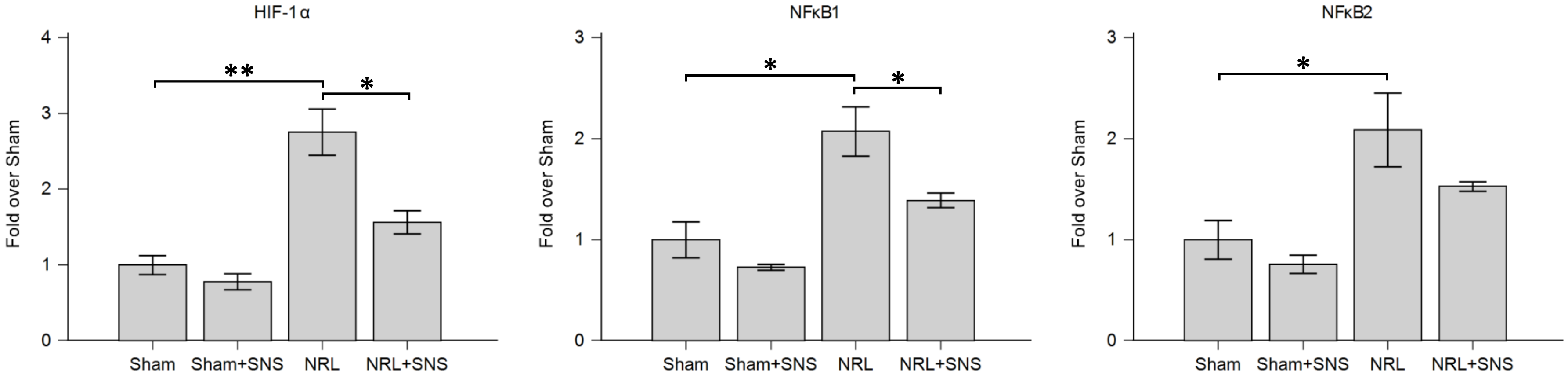


**Supplementary figure S7** The mRNA levels of genes associated with activated satellite glial cell and neuropathic pain quantified by nanostring nCounter assay.
